# Multi-omics analyses related to unfolded protein response in prostate cancer implicate pro-tumor role of IFRD1

**DOI:** 10.3389/fimmu.2026.1744197

**Published:** 2026-01-29

**Authors:** Yifeng Xue, Enyao Huang, Caichen Luo, Yanrong Qian, Tiange Wu, Chunyan Chu, Fan Shi, Shengrong Chen, Dakun Zhang, Weijun Chen, Weihua Huang, Ping Wang, Huixing Chen, Yifei Cheng, Yunxia Fan

**Affiliations:** 1 Department of Urology, Affiliated Jintan Hospital of Jiangsu University, Changzhou, China; 2 Department of Urology Affiliated Zhongda, School of Medicine, Zhongda Hospital, Southeast University, Nanjing, Jiangsu, China; 3 School of Medicine, Southeast University, Nanjing, Jiangsu, China; 4 Department of Pathology, School of Medicine, Zhongda Hospital, Southeast University, Nanjing, Jiangsu, China; 5 Urologic Medical Center, Shanghai General Hospital, Shanghai Jiao Tong University School of Medicine, Shanghai, China

**Keywords:** IFRD1, prognostic biomarker, prostate cancer, scRNA-seq, tumor microenvironment, UPR

## Abstract

**Introduction:**

The unfolded protein response (UPR) promotes prostate cancer (PCa) progression, yet its multi-omics landscape and clinical utility remain undefined.

**Methods:**

We integrated single-cell and bulk transcriptomic datasets, and identified UPR-related genes (UPRRGs) through a combination of differential expression analysis and weighted gene co-expression network analysis (WGCNA), based on which we further developed a consensus UPR-related signature (UPRRS) using a machine learning framework. The UPRRGs were further characterized by functional enrichment, cell-cell communication, and survival analyses. A clinically applicable nomogram integrating UPR-related prognostic genes was constructed for prognostic prediction. Through in silico and in vitro analyses, we validated the clinical relevance between the hub UPRRGs and PCa progression.

**Results:**

Single-cell analyses revealed elevated UPR activity in prostate epithelial cells, most prominently within the LE-KLK3 subpopulation. These cells exhibited enhanced ligand–receptor interactions in TNF, VEGF and NOTCH signaling axes. A seven-UPRRG signature (including IFRD1, DDIT3, HSPA5) demonstrated robust prognostic performance in the TCGA training set and three external validation cohorts (C-index > 0.82; AUC > 0.80). Multivariate Cox analysis confirmed UPRRS as an independent prognostic factor beyond clinical stage and Gleason score. Mechanistically, the UPRRS-high subgroup displayed an immunosuppressive microenvironment and reduced sensitivity to multiple chemotherapeutics. In vitro knock-down of IFRD1 markedly attenuated PCa cell proliferation and migration.

**Conclusion:**

We provide the first systematic single-cell atlas of UPR heterogeneity in PCa and develop a clinically translatable UPRRS prognostic model. IFRD1, a key driver, emerges as a dual diagnostic and therapeutic target, offering both theoretical and experimental foundations for precision stratification and individualized management of PCa.

## Graphical Abstract

**Figure:**
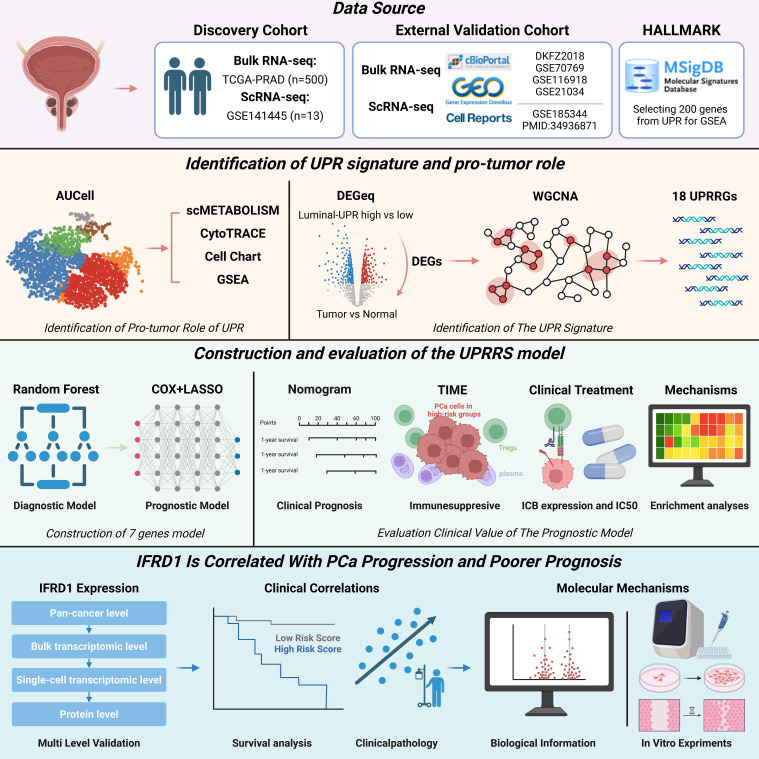

## 1 Introduction

Prostate cancer (PCa) remains a leading cause of cancer-related morbidity and mortality among men worldwide (1, 2). The development of castration-resistant prostate cancer (CRPC) represents a major therapeutic challenge in its management (3). Recently, the unfolded protein response (UPR) - an evolutionarily conserved pathway activated by endoplasmic reticulum (ER) stress - has been identified as a pivotal regulator of PCa progression, metastasis, therapeutic resistance, and immune evasion (4, 5). Under stress conditions such as hypoxia, nutrient deprivation, and therapeutic insult, the accumulation of misfolded proteins activates the UPR to restore protein homeostasis balance (6). However, in PCa, this adaptive response is frequently co-opted to promote tumor survival, metastasis, and treatment resistance, thereby presenting a promising therapeutic target. It remains unclear how UPR orchestrates PCa pathogenesis at a multi-omics level, integrating single-cell resolution, bulk transcriptomics, and clinical outcomes to establish a predictive framework.

The UPR is orchestrated by three principal sensor - IRE1α, PERK, and ATF6 - each of which initiates a distinct signaling cascade (6–8). In PCa, these UPR pathways are frequently upregulated and their activity correlates with advanced disease stage, high Gleason score, and poor clinical prognosis (9). Specifically, the IRE1α-XBP1 axis supports PCa cell survival under androgen-deprived conditions, whereas the PERK-eIF2α-ATF4 signaling pathway facilitates adaptation to oxidative stress (6). Furthermore, emerging evidence underscores the role of the UPR in remodeling the tumor microenvironment (TME), primarily via immunosuppressive mechanisms that impede T-cell infiltration and promote macrophage polarization (9, 10).

Therapeutic targeting of the UPR is gaining considerable momentum. Preclinical studies have demonstrated that pharmacological inhibition of IRE1α (e.g., using ORIN1001) not only suppresses tumor growth but also synergizes with immune checkpoint blockade (ICB) (11, 12). Conversely, hyperactivation of the UPR induced by compounds such as saikosaponin D (SSD) triggers lethal ER stress, thereby presenting a paradoxical strategy to eliminate cancer cells (13). Concurrently, clinical investigations are exploring UPR modulation in combination with other agents, such as PARP inhibitors (e.g., talazoparib) and androgen receptor antagonists (14). Despite these promising advances, several challenges persist, including pathway redundancy, context-dependent UPR outputs, and a lack of reliable biomarkers for patient stratification.

This study comprehensively characterizes the UPR landscape in PCa using a multi-omics approach. Leveraging integrated single-cell and bulk transcriptomic data, we first identified a set of UPR-related genes (UPRRGs) and subsequently constructed a consensus UPR-related signature (UPRRS) through a novel computational framework. We then rigorously evaluated the prognostic utility of UPRRS, demonstrating its robust ability to predict tumor incidence and disease progression. Furthermore, we developed a UPRRS-integrated nomogram designed to serve as a quantitative and clinically applicable tool for individualized prognosis prediction. To elucidate the underlying mechanisms, we performed multi-faceted analyses across bulk transcriptomic, genomic, and single-cell transcriptomic dimensions. These analyses revealed significant associations between UPRRS and key clinical outcomes in PCa patients.

## 2 Methods

### 2.1 Data source

The RNA sequencing (RNA-seq) data and clinical information of PRAD samples were obtained from the UCSC Xena database(https://xenabrowser.net/datapages/) (15). To identify PRAD patient samples within the TCGA cohort, we followed the screening methodology outlined in a previous PRAD-related study, excluding samples with incomplete survival data (16). Ultimately, we identified 496 PRAD samples and 52 adjacent normal tissue samples. We also obtained RNA-seq data and clinical characteristics from a cohort of PRAD patients, sourced from the GSE141445 dataset available in the Gene Expression Omnibus (GEO) (https://www.ncbi.nlm.nih.gov/geo/) (17). Single-cell RNA sequencing (scRNA-seq) data from GSE185344 (18) and Cell Reports (PMID: 34936871) (19) verified our results. The TCGA-PRAD, GSE116918 (20), and GSE21034 (21), GSE70769 (22) and DKFZ2018 datasets were utilized for the external validation of the IFRD1 gene. The transcriptomic and clinical data for DKFZ2018 were obtained from the cBioportal database (https://www.cbioportal.org/datasets) (23). These datasets were also employed to assess IFRD1 expression and conduct prognostic analyses. Additionally, we utilized the BEST tool (https://rookieutopia.hiplot.com.cn/app_direct/BEST/) to analyze and download multiple PCa expression and survival datasets, which served as external cohorts for IFRD1 expression and prognostic analysis. Single-cell RNA sequencing (scRNA-seq) reads were log-normalized in Seurat; genes detected in <3 cells, cells with <200 or >7–000 genes, or >10% UPR reads were removed. Batch effects were corrected with Harmony, clusters were identified by canonical markers and visualized with t-SNE. UPR activity was calculated with AddModuleScore; Differentially expressed genes (DEGs) between UPR-high and UPR-low luminal cells (Wilcoxon, adj-P < 0.05) were carried forward.

### 2.2 Single-sample gene set enrichment analysis

To quantify the activity of a gene set in each individual tumor, we first derived per-sample UPR scores by applying single-sample gene-set enrichment (ssGSEA) implemented in the GSVA R package to the TCGA-PRAD cohort. Next, we expanded the same ssGSEA framework to the 50 MSigDB hallmark gene sets, generating a pathway-activity matrix. Differential pathway activity between tumors stratified into UPR-high and UPR-low groups was then assessed with limma, and significance was defined after multiple-testing correction.

### 2.3 Weighted gene co-expression network analysis

A scale-free co-expression network (soft power = 6) was built from merged single-cell/bulk DEGs; the module most correlated with UPR (MEturquoise) was retained and its hub genes extracted.

### 2.4 Construction of prognostic signature by integrative machine learning approaches

Intersected DEGs were filtered by univariate Cox (P < 0.05), random-forest importance (MDA/MDG) and Least Absolute Shrinkage and Selection Operator (LASSO) (10-fold cross-validation) to yield seven UPR-related prognostic genes. UPRRS = Σ (expression × β) dichotomized patients at the median; performance was assessed with time-dependent Receiver Operating Characteristic (ROC) and log-rank tests.

### 2.5 Construction of UPR-related signature

Seven UPRRGs associated with PFS were identified by log-rank screening (P < 0.05); four remained after univariate Cox (P < 0.05) and random-forest confirmation. LASSO (10-fold cross-validation) converted these into a weighted index (UPRRS = Σ Ei × βi). Patients were split at the median; ROC and Kaplan–Meier (log-rank, P < 0.05) assessed discrimination.

### 2.6 Construction and evaluation of predictive nomogram

Considering that clinicopathological variables significantly influence survival outcomes, we constructed a nomogram utilizing TCGA clinicopathological parameters alongside UPRRS to enhance the predictive accuracy of UPRRS. Clinicians can leverage the points derived from the nomogram to estimate the patients’ survival probabilities. Calibration curves and ROC curves were generated, and the area under the curve (AUC) was calculated to assess the predictive capability of the nomogram.

### 2.7 Comprehensive analysis of immune characteristics and immune checkpoint inhibitor therapy response

To relate the UPRRS to the immune landscape of PCa, we first deconvoluted bulk RNA-seq profiles with Cell-type Identification by Estimating Relative Subsets of RNA Transcripts (CIBERSORT) to obtain relative fractions of 22 infiltrating immune cell types. The robustness of these estimates was confirmed in parallel with ESTIMATE, ssGSEA and XCell. The anti-cancer immune cycle is a crucial component of tumor immunotherapy, which involves several steps to constitute the anti-cancer immune cycle. Finally, immunogenicity was quantified with the Immunophenoscore (IPS) – an integrative, machine-learning metric that incorporates expression signatures of immunomodulators, immunosuppressive cells, MHC molecules and effector cells; high IPS predicts greater benefit from immune-checkpoint blockade. IPS values for TCGA-PRAD tumors were downloaded from The Cancer Immunome Atlas (TCIA).

### 2.8 Significance of the UPRRS in drug sensitivity

The drug sensitivity training data were downloaded from the Genomics of Drug Sensitivity in Cancer (https://www.cancerrxgene.org) (24). Subsequently, these data were validated using TCGA data to identify commonly used anti-tumor drugs for our analysis. The results were effectively presented through grouped comparison plots utilizing the “oncoPredict” and “ggpubr” packages. The Wilcoxon test was employed to ascertain the statistical differences between various groups, with a *p-value* < 0.05 considered statistically significant.

### 2.9 Pan-cancer analysis

We utilized the ‘TCGAplot’ package to conduct a comprehensive pan-cancer analysis and visualization of multi-omics data related to IFRD1 within TCGA. The source code and pre-built package are available on GitHub (https://github.com/tjhwangxiong/TCGAplot) (25).

### 2.10 Real-time quantitative reverse transcription polymerase chain reaction

Cellular RNA was isolated with the RNAeasy™ Animal RNA Isolation Kit (Beyotime, Shanghai) according to the vendor’s spin-column protocol. Complementary DNA was synthesized and subsequently amplified on a real-time PCR instrument with Taq Pro Universal SYBR qPCR Master Mix (BestEnzymes, Lianyungang). Gene-specific primers for IFRD1 and the housekeeping gene GAPDH were purchased from GENERAL BIOL (Chuzhou). Knockdown experiments were performed with 3 biological replicates, and qPCR was conducted in quadruplicate for each sample (4 technical replicates).

### 2.11 Clinical specimens and immunohistochemistry

For the internal cohort, Paraffin-embedded prostatectomy specimens (Sept–Dec 2024, Zhongda Hospital) were stained for IFRD1 (ProteinTech) by the hospital pathology unit; expression was scored semi-quantitatively. IRB approval: 2023ZDSYLL056-P01. For the independent external cohort, protein expression levels were assessed using publicly available IHC data retrieved from the Human Protein Atlas (HPA, https://www.proteinatlas.org) database. Representative IHC staining images were selected from PRAD tissue samples, including two high-grade and two low-grade tumors. The staining intensity and subcellular localization patterns were qualitatively evaluated for each sample to compare IFRD1 protein expression between disease aggressiveness categories.

### 2.12 Wound healing assay and plate cloning experiment

Wound-healing: si-IFRD1 or control PCa cells were grown to confluence in 6-well plates, scratched with a 200 µL tip, washed, and tracked in serum-free medium at 0, 12, 24, 48 h.

Colony formation: 1 × 10³ cells/well were seeded, fed every 3 days, fixed (4% PFA, 30 min) and stained (0.1% crystal violet, 30 min) on day 7–14 before counting.

### 2.13 Statistical analysis

Analyses were performed in R 4.1.2/3.6.3. Chi-square or Wilcoxon tests compared clinical variables; DEG significance was false discovery rate (FDR)-adjusted. Survival was assessed by Kaplan–Meier/log-rank and Cox models; ROC-AUC used timeROC. Immune correlations were evaluated with Spearman. qRT-PCR and two-group comparisons used Student’s t-test. *P < 0.05 was considered significant.

## 3 Results

### 3.1 UPR landscape in PCa at single-cell resolution

**Figure 1:**
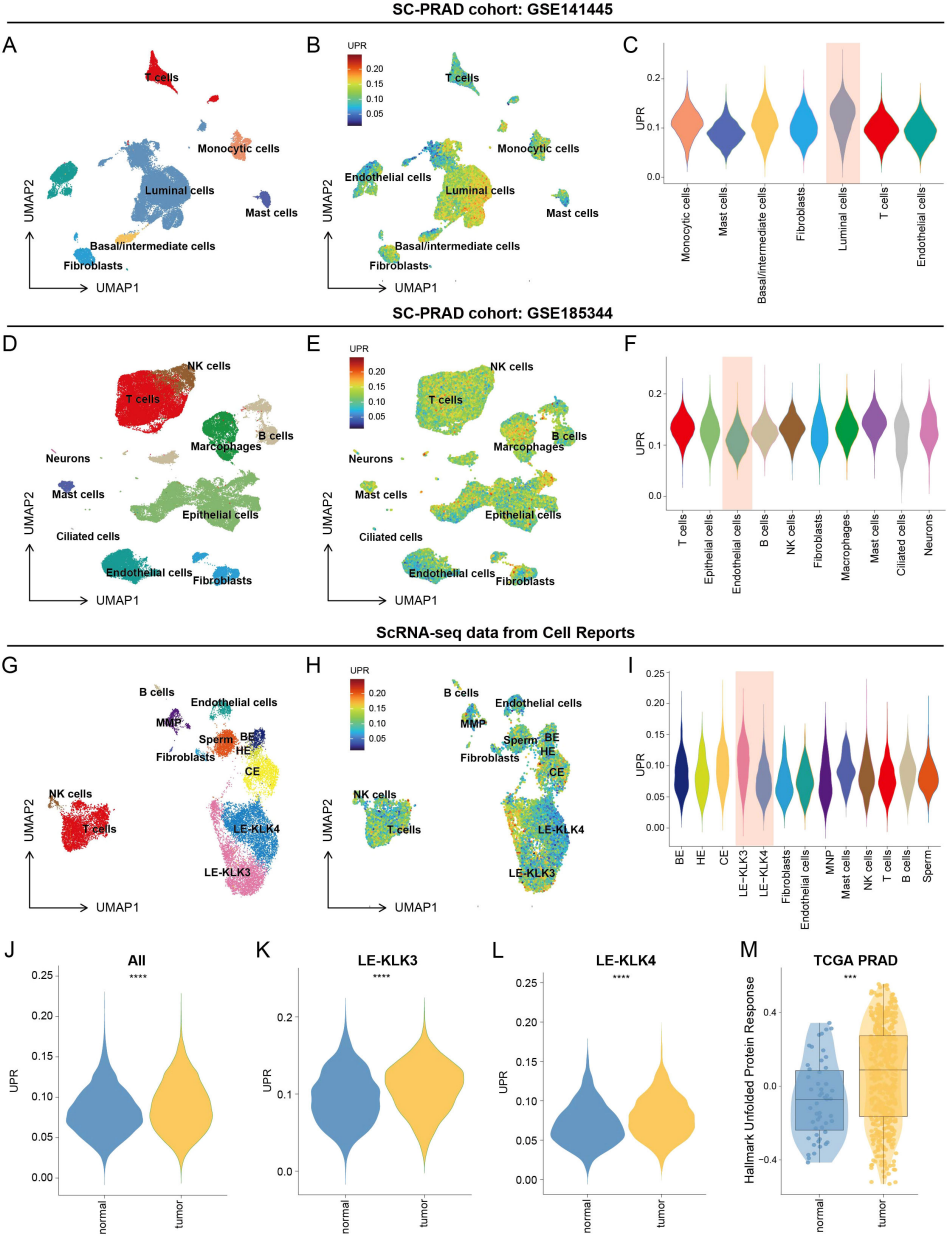
Single-cell transcriptomes identify UPR characterization within PCa luminal cells. **(A–C)** In the GSE141445 cohort, UMAP plot **(A)** showing the seven cell types identified by marker genes. UMAP plot **(B)** showing the expression level of UPR in each cell. Violin plot **(C)** showing the distribution of the UPR activity in different cell types. **(D–F)** In the GSE185344 cohort, UMAP plot **(D)** showing the ten cell types identified by marker genes. UMAP plot **(E)** showing the expression level of UPR in each cell. Violin plot **(F)** showing the distribution of the UPR activity in different cell types. **(G–I)** In the scRNA-seq data from Cell Reports, UMAP plot **(G)** showing the thirteen cell types identified by marker genes. UMAP plot **(H)** showing the expression level of UPR in each cell. Violin plot **(I)** showing the distribution of the UPR activity in different cell types. **(J–M)** Violin plot showing the expression levels of DEGs between normal population and tumor patients in all cell types **(J)**, LE-KLK3 **(K)**, LE-KLK4 **(L)**, and in the TCGA-PRAD database **(M)** respectively. ***p < 0.001, ****p < 0.0001.

In the study, we utilized publicly available scRNA-seq data (GSE141445) to clustering analysis (17, 26). Cell clusters were subsequently annotated into seven major cell type - monocytes, mast cells, basal/intermediate cells, fibroblasts, luminal cells, T cells, and endothelial cell - based on canonical marker genes (**Figure 1A**). Data from seven samples across different batches were integrated to mitigate batch effects, as visualized in **Supplementary Figure S1A**. Each cell type was characterized by a distinct molecular fingerprint (**Supplementary Figure S1B**). A heatmap further illustrates the top five marker genes defining each cluster (**Supplementary Figure S1C**). To quantify UPR activity across distinct cell types, we applied the “AddModuleScore” function in Seurat to calculate a UPR gene set enrichment score for each cell (**Figure 1B**). Among the seven identified cell types, luminal cells exhibited significantly elevated UPR activity compared to others (**Figure 1C**). Based on this UPR activity metric, luminal cells were stratified into UPR-high and UPR-low groups. Differential expression analysis between these two groups identified key genes (DEGs) for subsequent investigation (**Supplementary Table S2**; **Supplementary Figures S2A–C**). To validate these findings, we analyzed another scRNA-seq dataset (GSE185344) (18). Consistent with our initial results, a subset of epithelial cells displayed UPR-high gene set expression in this cohort (**Figures 1D–F**). Furthermore, leveraging an additional scRNA-seq dataset (19), we sub-classified luminal epithelial cells into LE-KLK3 and LE-KLK4 subtypes. We observed distinct UPR activity between these subtypes, a finding that corroborates the GSE185344 data. Specifically, UPR activity was significantly higher in the LE-KLK3 subtype compared to the LE-KLK4 subtype (**Figures 1G–I**). The identified DEGs were upregulated in tumor patients at the bulk transcriptome level, particularly within the LE-KLK3 subtype. Notably, these genes demonstrated significant utility in distinguishing tumor from non-tumor samples in the TCGA-PRAD cohort (**Figures 1J–M**). To strengthen the clinical relevance of UPR activation, we evaluated its association with standard prognostic indicators. UPR scores showed significant positive correlations with Gleason grade, pathological T stage, and PSA levels, and robustly predicted both PFI and OS in multiple cohorts, underscoring its potential as a clinically actionable pathway (**Supplementary Figures S1D, E**).

### 3.2 Identification of the regulator role of UPR in PCa

**Figure 2:**
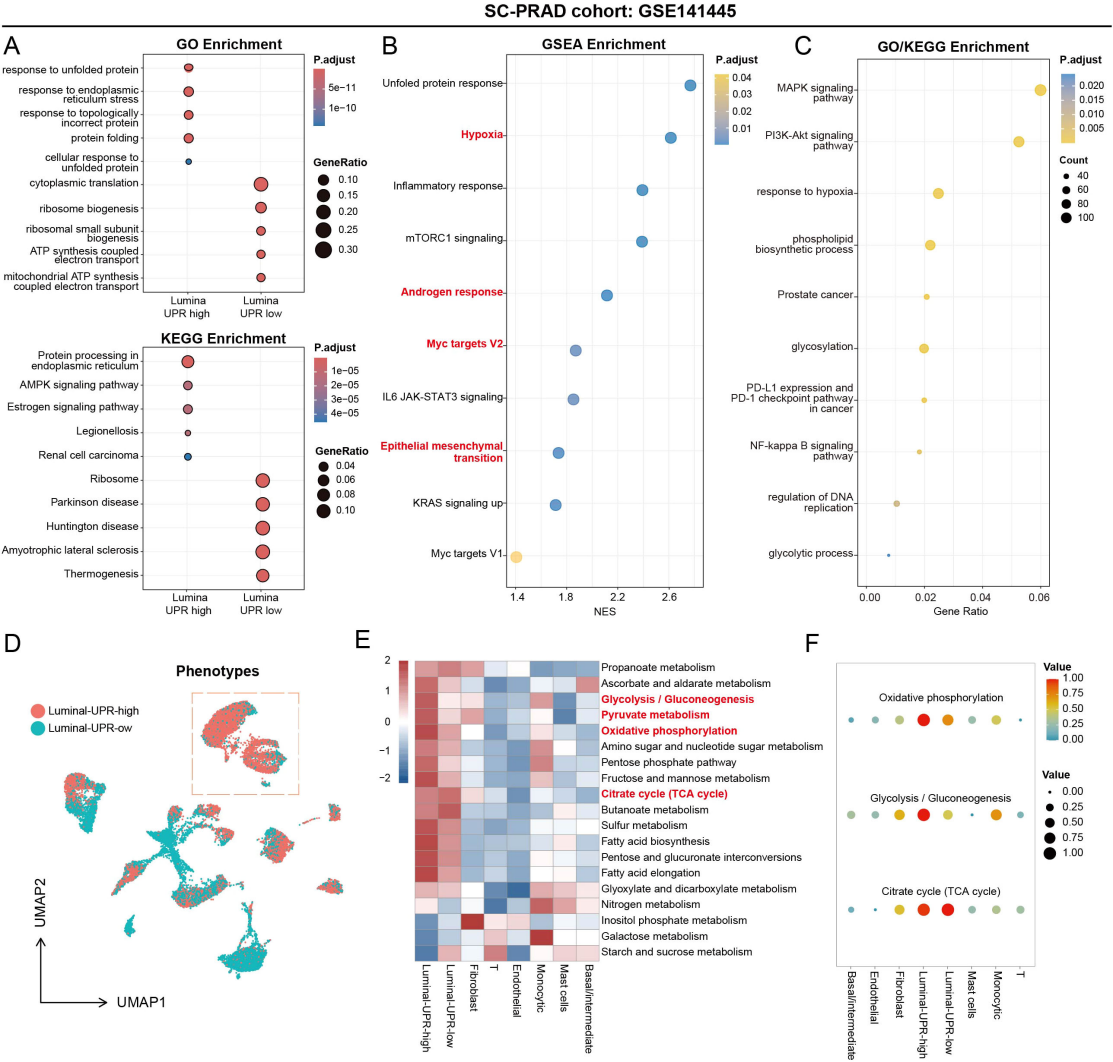
UPR activity drives transcriptomic divergence in PCa epithelial cells. **(A)** GO and KEGG functional enrichment analysis for DEGs between high-UPR and low-UPR luminal cells in the GSE141445 cohort. **(B)** GSEA functional enrichment analysis in the UPR-high luminal cells in the GSE141445 cohort. **(C)** GO and KEGG functional enrichment analysis for DEGs in the UPR-high luminal cells in the GSE141445 cohort. **(D)** UMPA plots showing the CytoTRACE analysis results regarding the phenotypes distribution between high- and low-UPR luminal cells in the GSE141445 cohort. **(E)** Heatmap showing the relationship between eight cell types and metabolic pathways. **(F)** Dot plot showing the correlation between the top three metabolic pathways for each cell type.

**Figure 3:**
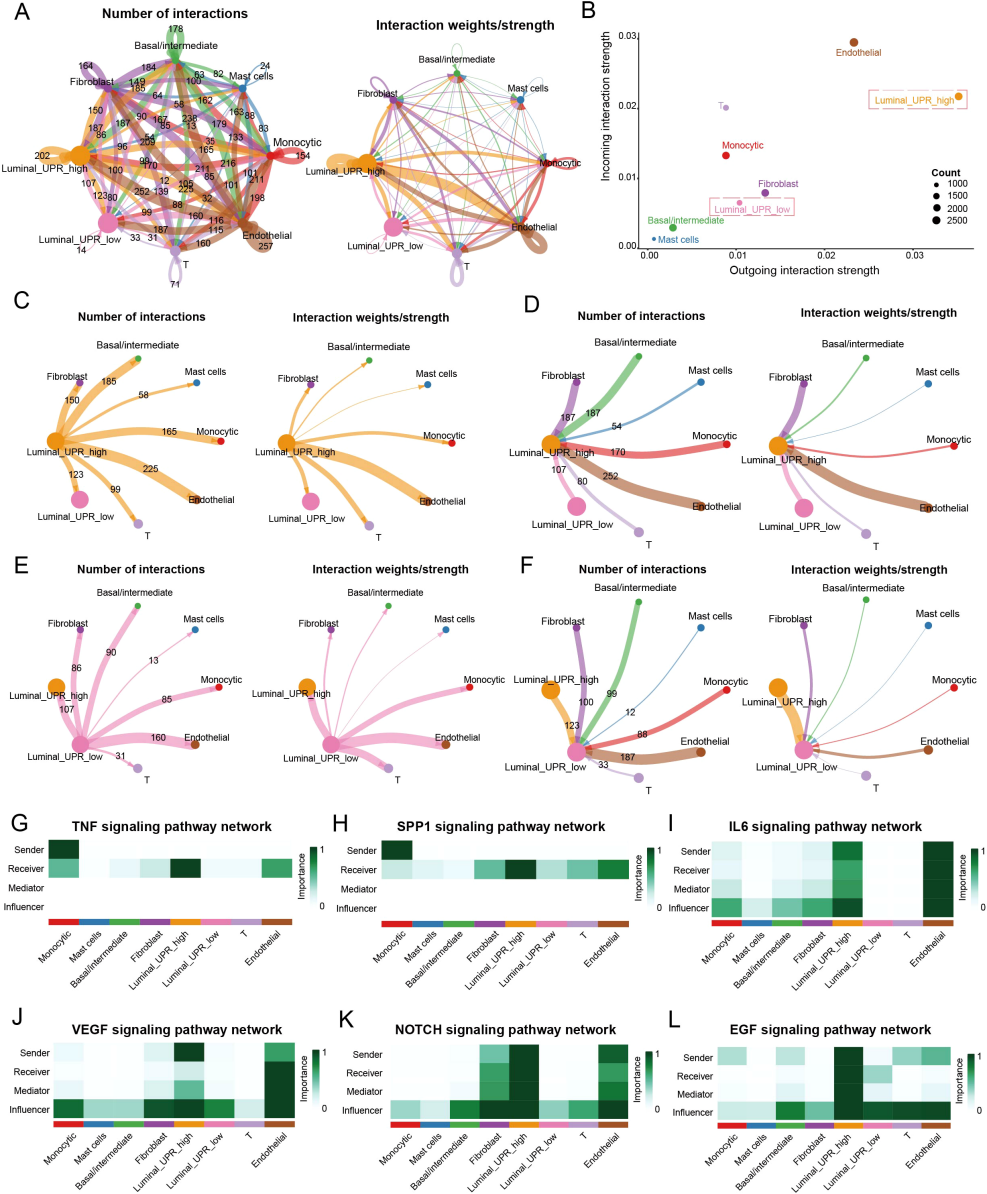
Cell-cell communications between cells types with different UPR activity. **(A)** Circos plots showing the cell networks. The size of each circle represents the number of cells in each cell cluster. ‘Number of interactions’ (left) indicates the number of ligand-receptor pairs that enable cell communication between two cell clusters; ‘Interaction weights/strength’ (right) represents the intensity of information exchange between two cell clusters. **(B)** The scatter diagram showing the incoming and outgoing interaction strength between all cell types. **(C, D)** Circos plots showing networks between other cell types and the luminal-UPR-high cells as the source **(C)**, as the target **(D–F)** Circos plots showing networks between other cell types and the luminal-UPR-low cells as the source **(E)**, as the target **(F–L)** Heatmaps showing the roles of different cell types playing in the TNF **(G)**, SPP1 **(H)**, IL-6 **(I)**, VEGF **(J)**, NOTCH **(K)** and EGF **(L)** pathway network.

To delineate the molecular programs downstream of UPR activation in PCa, we conducted functional enrichment and trajectory inference analyses comparing UPR-high and UPR-low luminal cells identified from the scRNA-seq dataset (GSE141445) (17). As shown in **Figure 2A**, Gene Ontology (GO) analysis revealed that the UPR-high luminal cell population was significantly enriched for terms related to the response to unfolded protein, ER stress, and response to topologically incorrect protein. In contrast, the UPR-low population was associated with processes including cytoplasmic translation, ribosome biogenesis, and mitochondrial ATP synthesis coupled electron transport, suggesting a bioenergetic and translational state distinct from ER stress adaptation (27). Kyoto Encyclopedia of Genes and Genomes (KEGG) pathway analysis further revealed that DEGs upregulated in UPR-high luminal cells were primarily enriched in pathways such as protein processing in ER, AMPK signaling pathway, and estrogen signaling pathway. Conversely, DEGs from the UPR-low group were enriched in ribosome, amyotrophic lateral sclerosis, and thermogenesis pathways. GSEA results revealed significant positive associations between the UPR DEG signature and hallmark gene sets including the UPR, hypoxia, inflammatory response, and androgen response (**Figure 2B**; **Supplementary Figure S2D**). These associations were further validated in the TCGA-PRAD cohort, where Myc targets v1 and v2 were among the most significantly enriched gene sets, implicating the UPR in proliferative and metabolic reprogramming (**Supplementary Figures S2F–H**) (28). Specifically, the correlations between the UPR DEGs and both the hypoxia and androgen response pathways were positive. Extended functional enrichment analysis of high-UPR luminal cells revealed broader biological consequences beyond protein homeostasis, including activation of MAPK and PI3K-Akt oncogenic pathways, metabolic reprogramming toward glycolysis, and upregulation of immune evasion signatures such as the PD-1/PD-L1 checkpoint axis (**Figure 2C**). CytoTRACE analysis showed that cellular developmental potential was not correlated with the UPR DEGs expression (**Figure 2D**; **Supplementary Figure S2E**), suggesting that UPR activation is not overtly linked to stemness or differentiation trajectories. Finally, we assessed metabolic pathway activities across cell types. Both UPR-high and UPR-low luminal cells were associated with oxidative phosphorylation, glycolysis/gluconeogenesis, and the citrate cycle (TCA cycle), indicating a coordinated metabolic rewiring to sustain ATP production and redox balance under ER stress (**Figures 2E, F**) (29). To investigate the role of UPR DEGs within the TME at single-cell resolution, we stratified luminal cells into UPR-high and UPR-low groups and systematically analyzed their intercellular communication networks. In the TME, distinct cell populations can function as senders, receivers, mediators, or influencers of specific signaling pathways, thereby shaping directed cellular crosstalk (30). Our analysis revealed that UPR-high luminal cells engage in communication with a broader repertoire of TME cells, particularly endothelial cells (**Figures 3A–F**), and function as more prominent senders, receivers, mediators, and influencers within key signaling pathways, including TNF, SPP1, IL6, VEGF, NOTCH, and EGF (**Figures 3G–L**). This prominent communicative role suggests that UPR-high luminal cells may critically regulate innate immune responses, inflammatory processes, tumor cell differentiation, migration, and ultimately, cancer cell survival. To support this notion, further analysis of the global signaling patterns identified UPR-high luminal cells as central hubs within both outgoing and incoming signaling networks (**Supplementary Figure S3A**). Their elevated connectivity within the TME underscores a role for the UPR that extends beyond cell-autonomous stress adaptation to include paracrine regulation of neighboring immune and stromal cells (31).

### 3.3 Identification of the hub module and genes related to UPR in bulk RNA-seq

**Figure 4:**
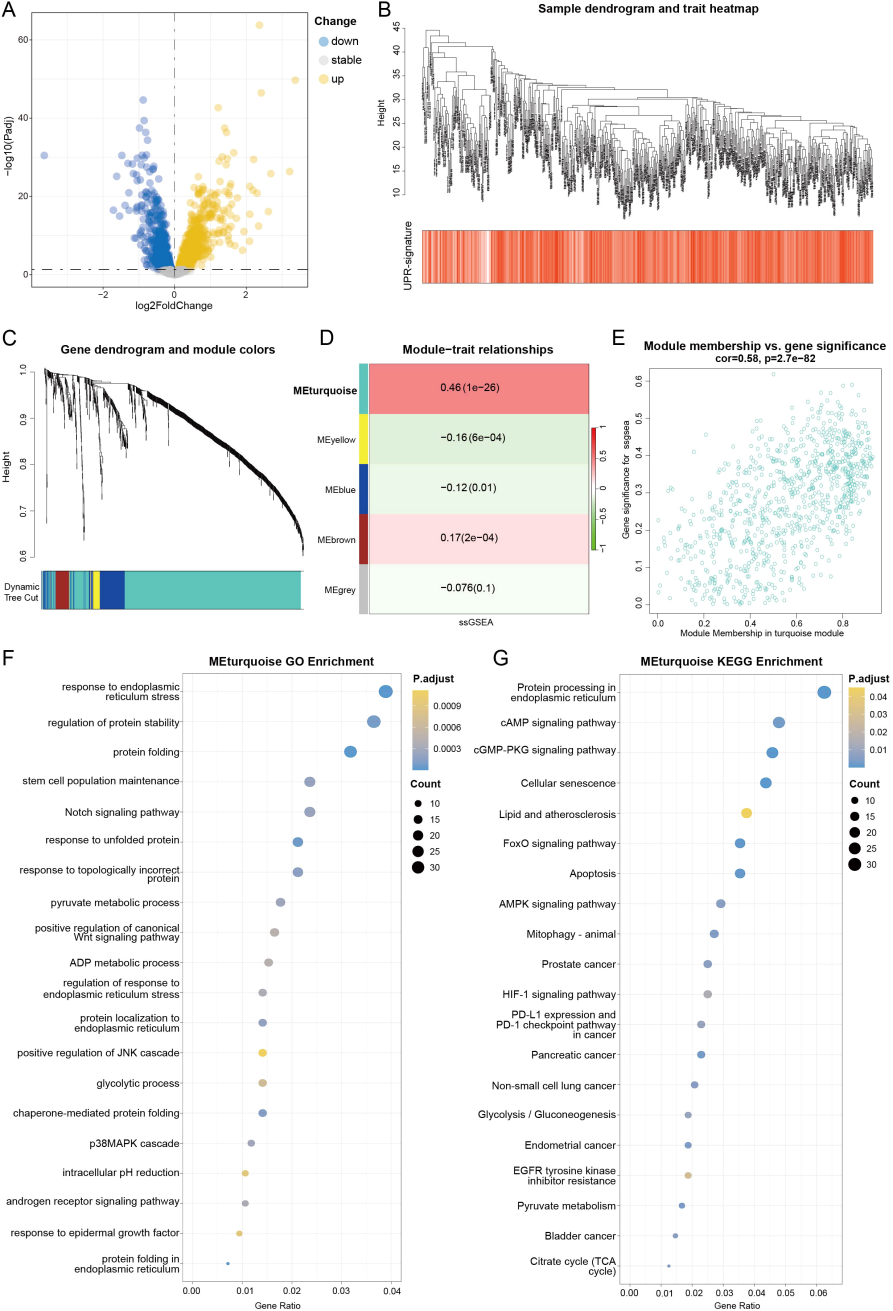
Identification of gene modules co-expressed with UPR among the DEGs using WGCNA. **(A)** Volcano plot showing the DEGs identified at the single-cell level with luminal-UPR-high versus luminal-UPR-low that were incorporated into the TCGA-PRAD cohort of tumor and normal tissue sample. **(B)** Dendrogram showing the hierarchical clustering of TCGA-PRAD samples based on UPR-signature. The bottom heatmap represents each sample’s UPR activity score, calculated by ssGSEA algorithm. **(C)** Cluster dendrogram of the WGCNA analysis. **(D)** Module-trait heatmap showing that the MEturquoise module was closely related to the UPR activity score. **(E)** Scatter plot showing the relationship between GS and MM in the blue module. **(F, G)** Bubble charts of GO **(F)** and KEGG **(G)** functional enrichment analysis of the genes within the Meturquoise module.

In this study, the UPR DEGs derived from single-cell analysis was consistently validated in bulk transcriptomes, confirming elevated UPR activity at the macroscopic tissue level. A volcano plot depicts the DEGs identified between UPR-high and UPR-low luminal cells from the scRNA-seq data, using thresholds of |logFC|>0.5 and p.adj<0.05 (**Figure 4A**). Then we quantified UPR activity for each sample in bulk transcriptomes using ssGSEA, which calculates the enrichment score of a predefined UPR gene set at the individual sample level. These UPR enrichment scores were subsequently employed as phenotypic traits for WGCNA (32). Following the removal of outlier samples and determination of an optimal soft-thresholding power (β = 6; **Supplementary Figure S3B**), a scale-free co-expression network was constructed. We then applied WGCNA to the TCGA-PRAD dataset to identify gene modules associated with UPR activity. The set of DEGs (logFC>0.5 and *p* < 0.05) derived from this single-cell analysis was utilized for co-expression network construction following the removal of outlier samples (**Figure 4B**). Hierarchical clustering identified five distinct gene modules (**Figure 4C**). Among these, the MEturquoise module exhibited the strongest positive correlation with UPR activity (cor = 0.45, p = 1e-26; **Figure 4D**). The functional relevance of the MEturquoise module to UPR was further corroborated by a strong positive correlation between gene significance (GS) for UPR and module membership (MM) within this module (cor = 0.58, *p* = 2.7e-82; **Figure 4E**; **Supplementary Table S3**), indicating that genes highly connected within the module are also central to UPR biology. A Topological overlap matrix (TOM) heatmap visually confirmed the high degree of co-expression connectivity among these UPRRGs within the module (**Supplementary Figures S3C, D**). Functional enrichment analysis of the UPRRGs revealed their significant involvement in biological processes and pathways directly related to UPR, including the response to ER stress, regulation of protein stability, and protein folding (**Figures 4F, G**).

### 3.4 Construction of diagnosis and prognosis signatures based on integrative machine learning

**Figure 5:**
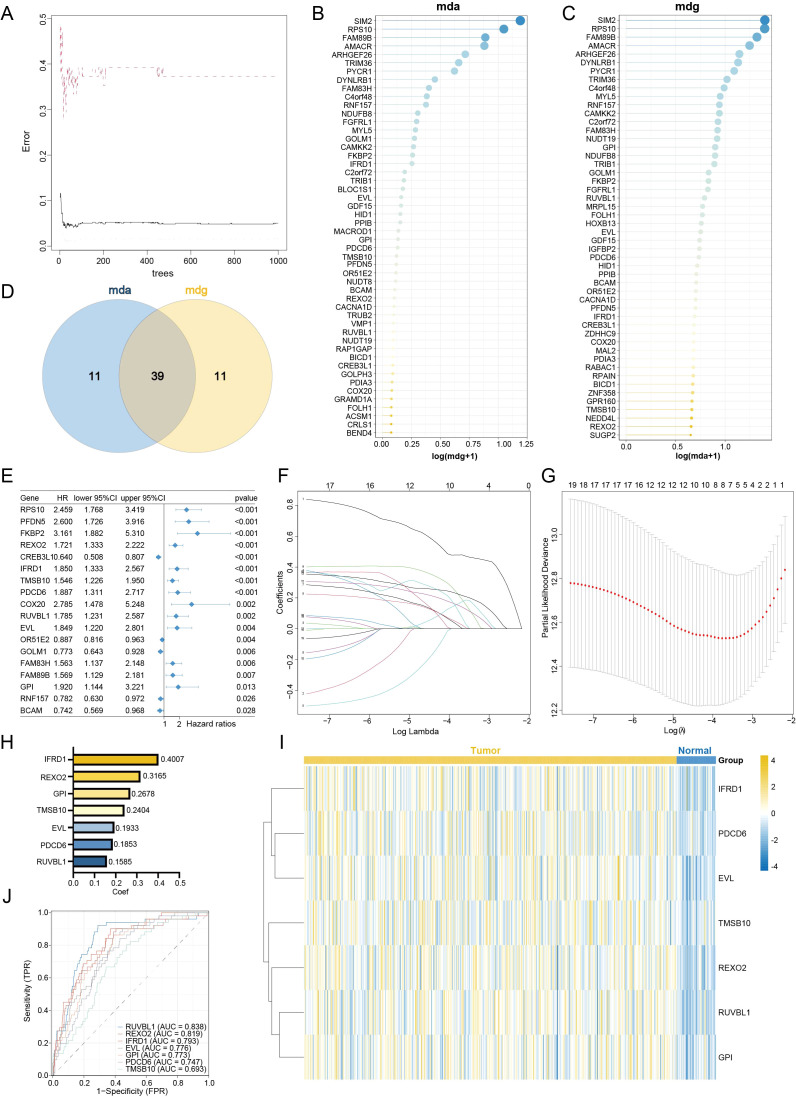
Construction of the UPR-related diagnosis and prognostic signature. **(A)** Random Forest Error Curve Chart. **(B, C)** Bar plot of log-rank test results for the top 50 genes from Random Forest analysis, with the x-axis representing log(mda+1) **(B)** and log(mdg+1) **(C, D)** A Venn diagram showing the overlap between DEGs determined by two random forest methods for evaluating feature importance in line with namely mda and mdg. **(E)** Forest plots showing the results of stepwise Cox regression. **(F, G)** Visualization of LASSO regression. The optimal λ was obtained when the partial likelihood deviance reached the minimum value. **(H, I)** Regression coefficients **(H)** of seven pivotal genes obtained in stepwise Cox regression, and heatmap **(I)** of the expression level in tumor and normal tissues for those seven genes. **(J)** ROC curves showing the specificity and sensitivity of UPRRS in predicting prognosis, implicating the diagnostic efficiency of UPRRS.

To construct a consensus UPRRS, we employed a multi-step machine learning framework to refine the genes within the MEturquoise module (32). An initial subset of 39 genes was selected for further modeling based on two criteria: significance (*p* < 0.05) in univariate Cox regression, and high importance assessed by both mean decrease accuracy (MDA) and mean decrease gini (MDG) in random forest analysis (**Figures 5A–D**; **Supplementary Table S4**). By intersecting the genes, we identified a core set of 18 genes, designated as UPRRGs, which are implicated in the UPR at both bulk-tissue and single-cell resolutions (**Figure 5E**). These genes were subsequently entered into the LASSO-Cox regression pipeline. The TCGA-PRAD cohort was randomly split into a training set and an internal validation set at a 1:1 ratio. Genes retaining non-zero coefficients from the LASSO regression were further refined using backward stepwise Cox proportional hazards regression, which yielded a final signature comprising eighteen genes. The optimal penalization parameter (λ = 0.023155) for the LASSO model was determined through tenfold cross-validation by minimizing the partial likelihood deviance (**Figures 5F–G**; **Supplementary Table S5**). The final UPRRS model was formulated based on the expression levels of seven pivotal genes: IFRD1, REXO2, GPI, TMSB10, EVL, PDCD6, and RUVBL1. In the training cohort, the coefficient for each gene in the signature was positively associated with an increased risk of poor progression-free interval (PFI). Among these seven genes, IFRD1 demonstrated the highest hazard ratio (HR = 1.85, 95% CI [1.33-2.57], *p* < 0.001; **Figure 5E**). All seven genes were markedly upregulated in tumor tissues compared to normal counterparts (**Figures 5H, I**; **Supplementary Figure S3E**). The prognostic power of each gene was quantitatively assessed using ROC curve analysis. The AUC for predicting poor prognosis were 0.838 (RUVBL1), 0.819 (REXO2), 0.793 (IFRD1), 0.776 (EVL), 0.773 (GPI), 0.747 (PDCD6), and 0.693 (TMSB10), confirming their significant individual predictive value (**Figure 5J**). To validate the diagnostic robustness of our LASSO-derived signature, we performed Random Forest analysis. The model confirmed IFRD1 as the most influential predictor thereby independently verifying the clinical relevance of the UPRRS (**Supplementary Figures S3F–G**).

### 3.5 Evaluation of the UPRRS model

**Figure 6:**
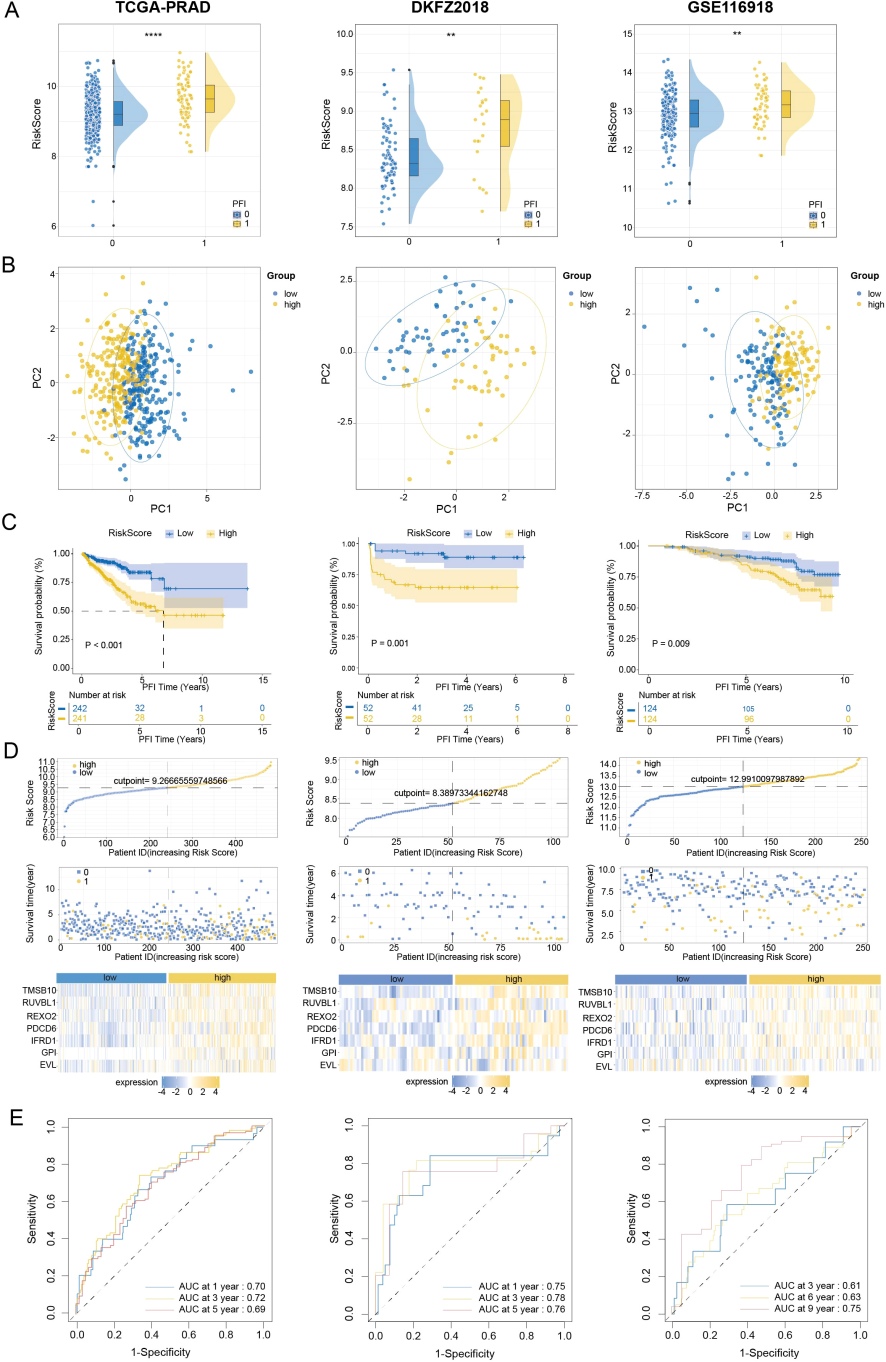
Evaluation the diagnostic and predictive efficiency of the UPRRS model. **(A)** Risk score of high and low PFI in the TCGA-PRAD, DKF2018, and GSE116918 cohort. **(B)** PCA of high and low expression of UPRRS in the TCGA-PRAD, DKF2018, and GSE116918 cohort. **(C)** Kaplan-Meier curves for PFI of PRAD patients stratified by median UPRRS in the TCGA-PRAD, DKF2018, and GSE116918 cohort. **(D)** Risk score nomograms depicting risk scores, survival time distribution, and expression of IFRD1, TMSB10, RUVBL1, REXO2, PDCD6, GPI and EVL in the TCGA-PRAD, DKF2018, and GSE116918 cohort. **(E)** Time-dependent ROC analysis for predicting 1-year, 3-year, and 5-year OS in PRAD patients in the TCGA-PRAD, DKF2018, and GSE116918 cohort. The left panels correspond to the TCGA-PRAD cohort, and the middle panels correspond to the DKF2018 cohort, and the right panels correspond to the GSE116918. **p < 0.01, ****p < 0.0001.

**Figure 7:**
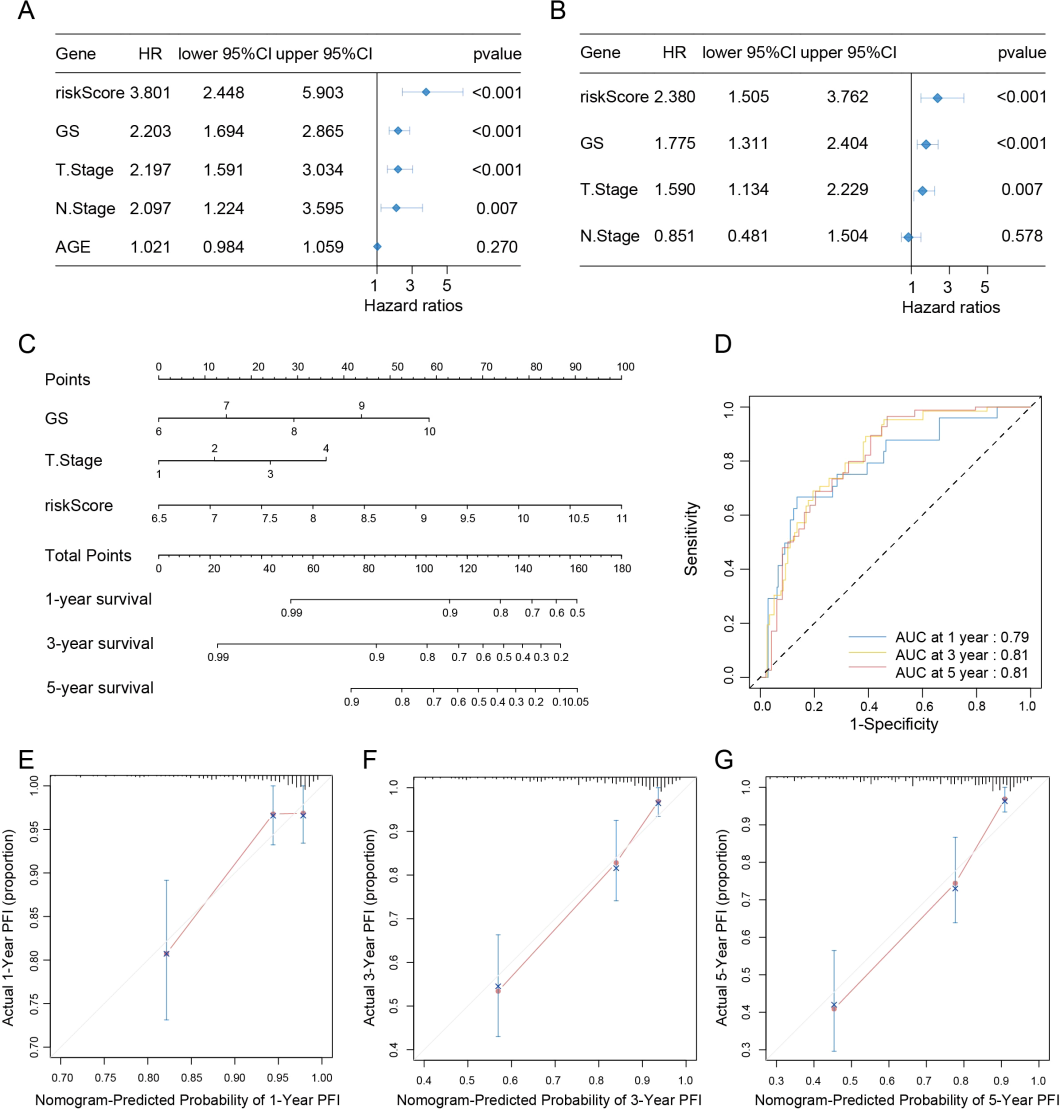
Construction of the nomogram for predicting OS in PCa patients. **(A, B)** Forest plot of univariate Cox regression analysis **(A)** and multivariate Cox regression analysis **(B)** results showing UPRRS and clinical features in PRAD patients. **(C)** Nomogram for predicting 1-year, 3-year, and 5-year OS in PRAD patients based on UPRRS, Gleason Score, T stage and risk score. **(D)** Time-dependent ROC analysis for predicting 1-year, 3-year, and 5-year OS in PRAD patients using the nomogram. **(E–G)** Calibration curves for predicting 1-year **(E)**, 3-year **(F)**, and 5-year **(G)** OS in PRAD patients based on the nomogram.

To establish the generalizability and clinical applicability of our model, we performed extensive validation across three independent cohorts, namely TCGA-PRAD, DKFZ2018 (23) and GSE116918 (20). Our results demonstrated that a higher UPRRS was significantly associated with an increased risk of PFI events. Principal component analysis (PCA) confirmed that the UPRRS effectively stratified patients into two distinct groups with significant prognostic separation in the TCGA-PRAD, DKFZ2018, and GSE116918 cohort. Consistent with this, patients classified into the UPRRS-high group exhibited a significantly higher risk score compared to those in the UPRRS-low group across all validation cohorts (**Figures 6A–C**). To facilitate clinical translation, we developed a PFI-specific nomogram that integrates the UPRRS to provide quantitative predictions of patient survival probabilities at 1, 3, and 5 years (**Figure 6D**). The robust predictive performance of the UPRRS was further validated by time-dependent ROC analysis. The model demonstrated consistent prognostic accuracy across multiple time points in the TCGA-PRAD cohort [AUC: 0.70 (1-year), 0.72 (3-year), 0.69 (5-year)], the DKFZ2018 cohort [AUC: 0.75 (1-year), 0.78 (3-year), 0.76 (5-year)], and the GSE116918 cohort [AUC: 0.61 (1-year), 0.63 (3-year), 0.75 (5-year)] (**Figure 6E**). To facilitate individualized survival estimation, we developed a clinically applicable nomogram that integrates the UPRRS with key clinical parameters. Univariate and multivariate Cox regression analyses consistently identified the UPRRS and T stage as the most robust independent predictors of overall survival (OS) in PCa (univariate HR = 3.801, multivariate HR = 2.380, both *p* < 0.001; **Figures 7A, B**). In contrast, age and N stage did not retain independent prognostic significance in the multivariate model. Consequently, the final nomogram incorporates only the UPRRS and clinical T (cT) stage to provide a practical tool for predicting survival probabilities in PCa patients (**Figure 7C**). The integrated nomogram exhibited superior predictive accuracy compared to the UPRRS alone, with time-dependent AUCs of 0.79 (1-year), 0.81 (3-year), and 0.81 (5-year) (**Figure 7D**). The calibration curves for 1-, 3-, and 5-year survival showed excellent agreement between nomogram predictions and observed outcomes (**Figures 7E–G**), confirming the model’s high reliability and clinical interpretability.

### 3.6 Seven predictive value genes have clinical value in subtyping clinical patients

**Figure 8:**
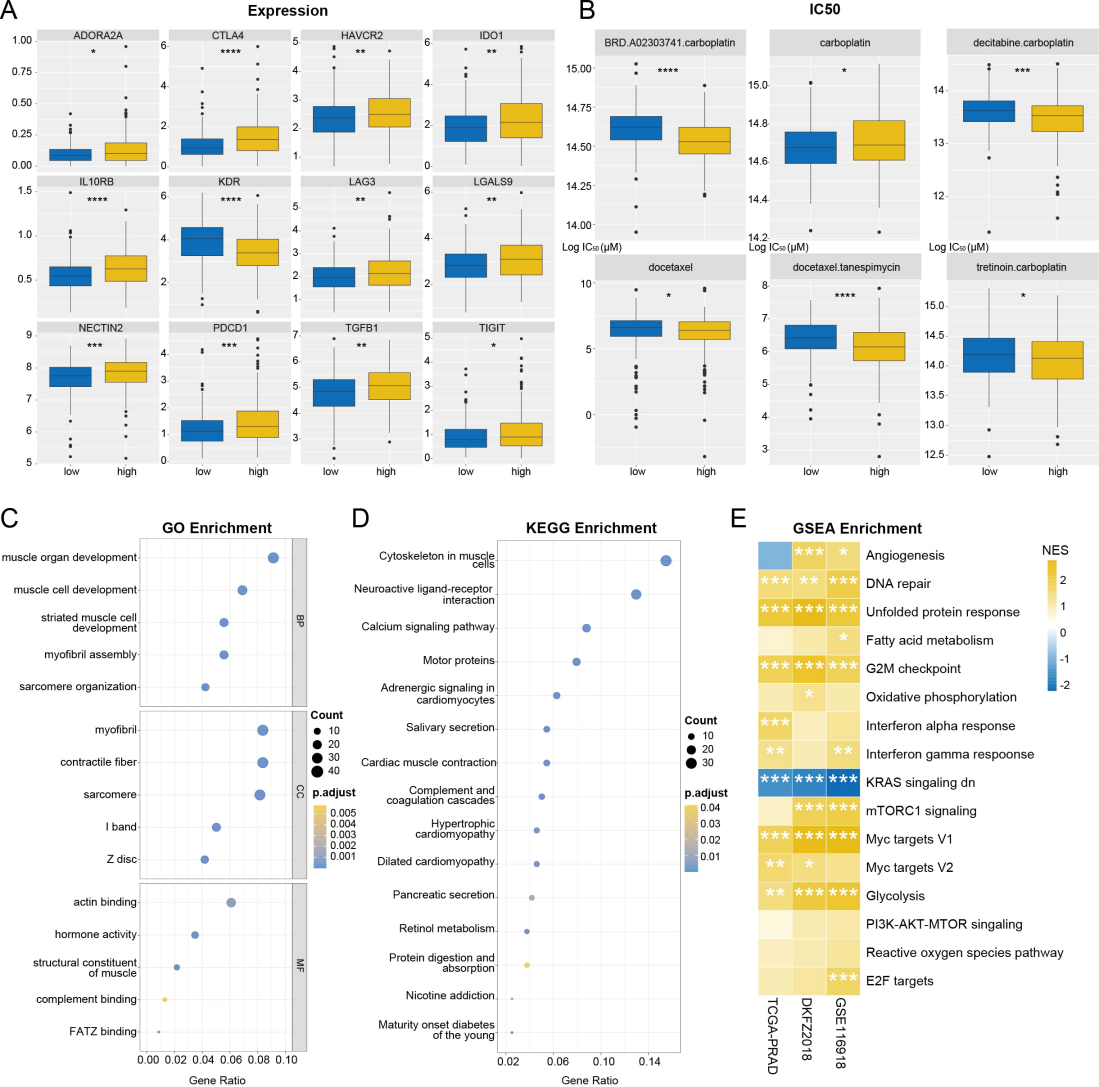
Clinical treatment correlation and mechanism analysis of UPRRS. **(A)** Expression of immune checkpoints in high- and low-risk groups. **(B)** IC50 values of six common clinical chemotherapeutic drugs in UPR-high and UPR-low groups. **(C, D)** Bubble chart of GO **(C)** and KEGG **(D)** functional enrichment analysis for UPRRS. **(E)** Heatmap showing correlation between the hallmark pathway activities and expression level of UPRRS in the TCGA-PRAD, DKF2018, and GSE116918 cohort respectively. *p < 0.05, **p < 0.01, ***p < 0.001, ****p < 0.0001.

The UPRRS stratified patients into distinct high- and low-risk groups, effectively capturing the underlying heterogeneity among them. Analysis of immune cell infiltration using Cell-type Identification by Estimating Relative Subsets of RNA Transcripts (CIBERSORT) revealed distinct landscape between the groups. The high-risk group was characterized by a higher abundance of regulatory T cells (Tregs) (**Supplementary Figures S4A, B**). We next investigated the relationship between UPR and immune activity by comparing the expression profiles of key immune checkpoint molecules between the two groups. Multiple immune checkpoints, including ADORA2A, CTLA4, HAVCR2, IDO1, IL10RB, LAG3, LGALS9, NECTIN2, PDCD1, TGFB1, and TIGIT, were significantly upregulated in the high-risk group, whereas KDR expression was notably lower (*p* < 0.05; **Figure 8A**). Furthermore, drug sensitivity analysis revealed that patients in the high-risk group exhibited increased sensitivity to several agents, including docetaxel, tanespimycin, and specific carboplatin-based combinations (e.g., BRD-A02303741-carboplatin, decitabine-carboplatin, tretinoin-carboplatin), but decreased sensitivity to carboplatin alone (**Figure 8B**). Functional enrichment analysis of the seven genes in UPRRS identified significant involvement in specific biological contexts, notably “cytoskeleton in muscle cells” and the “Neuroactive ligand-receptor interaction” pathway (**Figures 8C, D**). Collectively, across all three cohorts, high-risk tumor tissues consistently exhibited co-upregulation of oncogenic and metabolic hallmarks coupled with UPR activation. This pattern highlights a critical protein homeostasis-bioenergetics axis that underpins aggressive PCa progression (**Figure 8E**).

### 3.7 IFRD1 is a key mediator of UPR-driven PCa progression and predicts poor clinical outcome

To pinpoint the key regulatory gene within the UPRRS, we analyzed the seven genes and identified IFRD1 as a central hub gene consistently prominent in both single-cell and bulk transcriptomic analyses. Given its significant correlation with poor prognosis, we systematically investigated the biological role of IFRD1 in PCa pathogenesis.

**Figure 9:**
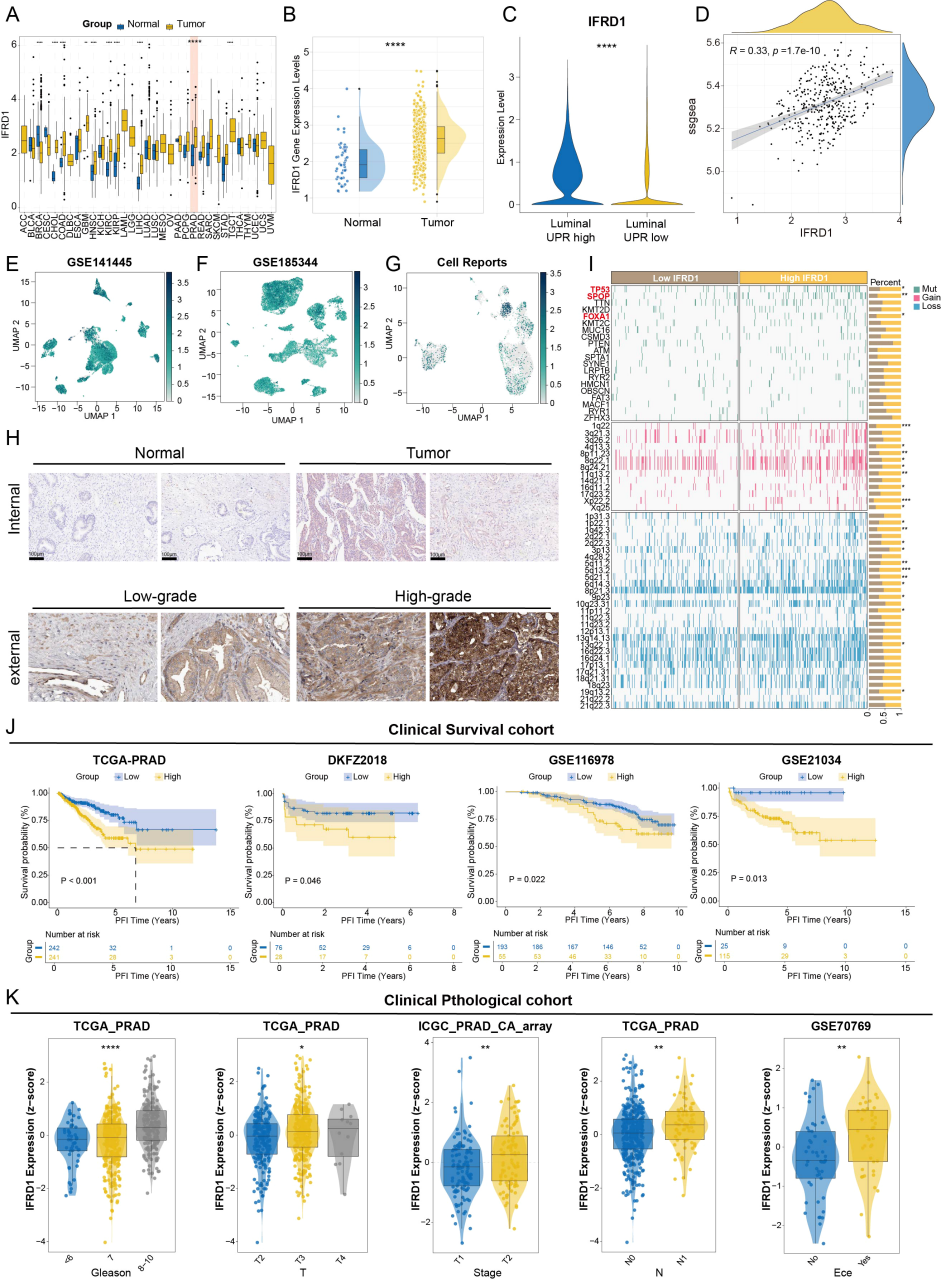
IFRD1 expression levels and its relationship with clinical outcomes. **(A)** Expression of IFRD1 in tumor and adjacent normal tissues across pan-cancer. **(B)** Violin plot showing the expression levels of IFRD1 in adjacent normal tissues and PRAD tumor tissues in the TCGA-PRAD cohort. **(C)** Violin plot showing the expression levels of IFRD1 in luminal-UPR-high and luminal-UPR-low cells in the GSE141445 cohort. **(D)** The linear correlation curves of the expression of IFRD1 and the ssGSEA score. **(E–G)** UMAP plots showing the expression levels of IFRD1 in single-cell level in the GSE141445 **(E)**, GSE185344 **(F)** cohort and the scRNA-seq data from Cell Reports **(G, H)** Representative images of IFRD1 protein IHC staining in tumor and adjacent normal tissues in both internal and external cohorts. **(I)** The waterfall plot of the somatic mutation landscape in UPR-high and UPR-low groups in mutation, gain and loss aspects respectively. **(J)** Kaplan-Meier curves for PFI of PRAD patients with high and low expression level of IFRD1 in the TCGA-PRAD, DKF2018, GSE116918 and GSE21034 cohort. **(K)** Box plots showing the clinical relevance of IFRD1 expression levels in the TCGA-PRAD, ICGC_PRAD_CA_array and GSE70769 cohort. *p < 0.05, **p < 0.01, ***p < 0.001, ****p < 0.0001.

**Figure 10:**
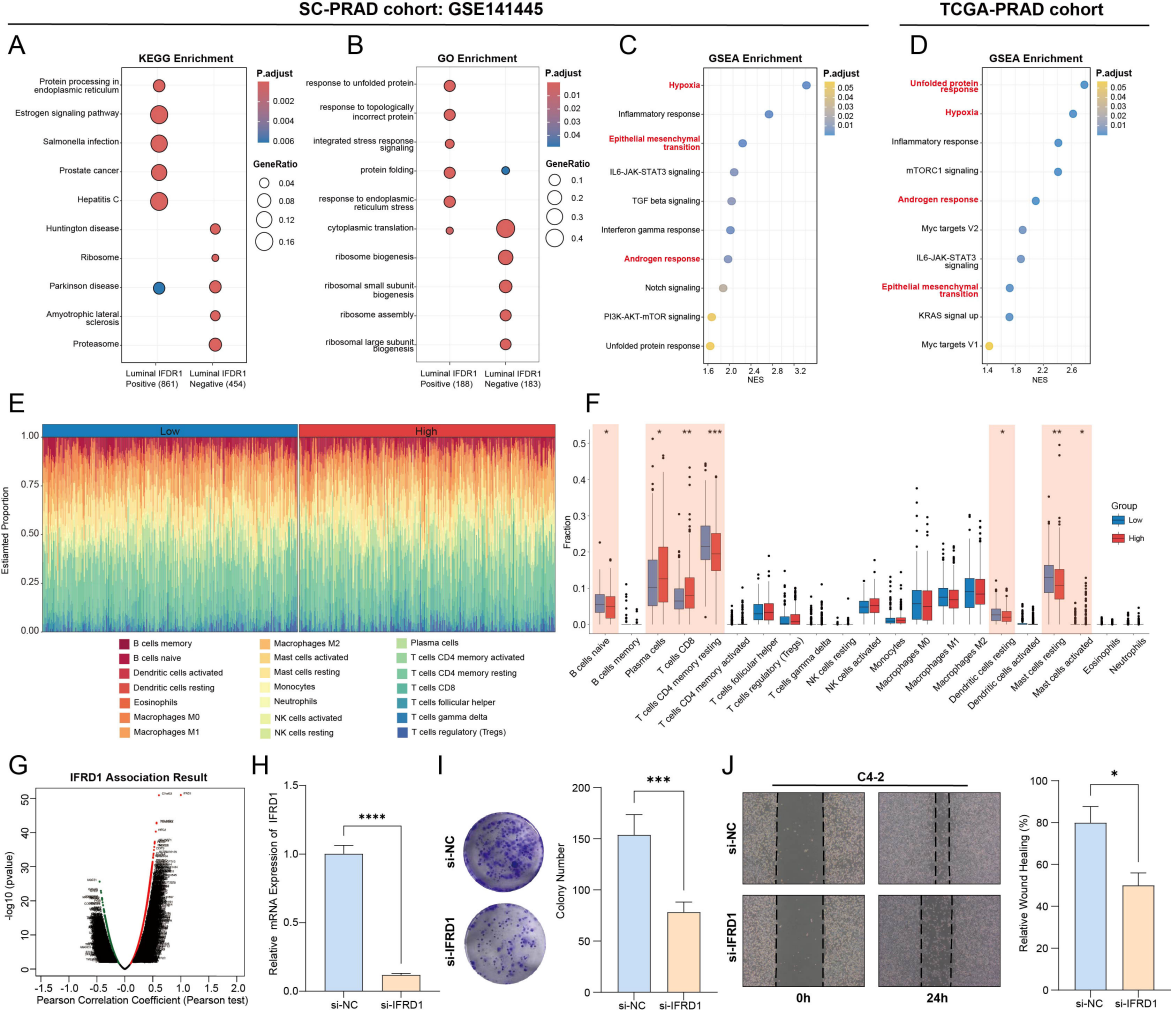
Potential mechanisms and biological experiments of IFRD1 in PRAD. **(A, B)** In the GSE141445 cohort, bubble charts of KEGG **(A)** and GO **(B)** functional enrichment analysis for DEGs between luminal-IFRD1-positive and luminal-IFRD1-negative subgroups. **(C)** GSEA functional enrichment analysis in the luminal-IFRD1-positive subgroup in the GSE141445 cohort. **(D)** GSEA functional enrichment analysis in the IFRD1-high groups TCGA-PRAD database. **(E)** Rainbow plot showing the proportions of 21 immune cell types calculated by CIBERSORT in high- and low-IFRD1 subgroups, with each bar representing a patient and each color representing an immune cell type. **(F)** Box plot showing the expression levels of immune cells between high- and low-IFRD1 subgroups. **(G)** Highly correlated genes of IFRD1 tested by Pearson test in TCGA-PARD cohort. **(H)** Efficiency of siRNA in inhibiting IFRD1 expression in C4–2 cells. RT-qPCR analysis was conducted using 3 biological replicates of knockdown samples, with each sample run in 4 technical replicates. **(I)** Colony formation assay to assess the impact of IFRD1 knockdown on cell proliferation. **(J)** Wound healing assay to evaluate the effect of IFRD1 knockdown on cell migration. *p < 0.05, **p < 0.01, ***p < 0.001, ****p < 0.0001.

Pan-cancer analysis initially revealed that IFRD1 was significantly upregulated in tumor tissues compared to adjacent normal tissues across multiple cancer types, including PCa (**Figure 9A**). In the TCGA-PRAD cohort, IFRD1 expression was confirmed to be markedly elevated in primary tumor tissues compared to normal controls (**Figure 9B**). Interestingly, at the single-cell resolution in the GSE141445 dataset, IFRD1 expression was significantly enriched in UPR-high luminal cells (**Figure 9C**). To validate the single-cell findings at the bulk tissue level, we examined the correlation between IFRD1 expression and UPR signature scores in the TCGA-PRAD cohort. A significant positive correlation (*R* = 0.33, *p* < 0.001) was observed, confirming that the IFRD1-UPR axis observed in individual cells is preserved in whole-tissue transcriptomes (**Figure 9D**). This expression pattern was consistently validated across two additional independent scRNA-seq datasets (GSE185344 and Cell Reports), where IFRD1 remained highly expressed in UPR-high luminal cells (**Figures 9E–G**). At the protein level, IHC staining from both internal and external cohort demonstrated substantially stronger IFRD1 expression in PCa tissues compared to matched normal tissues (**Figure 9H**). To explore the genomic alterations associated with IFRD1-mediated UPR activation, we compared the somatic mutation landscapes between the UPR-high and UPR-low groups. This analysis revealed distinct mutation patterns, with the UPR-high group exhibiting a higher mutational burden in key driver genes such as TP53, SPOP, and FOXA1. This finding suggests a potential mechanistic link between IFRD1-mediated UPR activation and genomic instability in PCa (**Figure 9I**). Kaplan–Meier survival analysis across multiple independent cohorts - TCGA-PRAD, DKFZ2018, GSE116918, and GSE21034 (21, 22) - consistently demonstrated that elevated IFRD1 expression was significantly associated with a shorter PFI, thereby confirming its robust prognostic value (**Figure 9J**). Furthermore, IFRD1 expression showed significant positive correlations with key indicators of disease aggressiveness, including advanced Gleason score, and higher T and N stages (**Figure 9K**). To elucidate the mechanisms underlying IFRD1-driven tumor progression, we performed functional enrichment analysis comparing luminal-IFRD1-positive and luminal-IFRD1-negative subgroups within the GSE141445 dataset. This analysis revealed that high IFRD1 expression was significantly enriched in pathways central to the UPR and ER stress, encompassing protein processing in the ER, ER-nucleus signaling, and estrogen response early pathways. In stark contrast, the IFRD1-low subgroup showed minimal enrichment for these UPR-related pathways, confirming IFRD1’s role as a critical upstream regulator that drives UPR activation, modulates protein homeostasis, and facilitates PCa progression. (**Figures 10A, B**). GSEA of the DEGs further associated IFRD1 with the activation of hallmark oncogenic pathways, including hypoxia, epithelial-mesenchymal transition (EMT), and androgen response (**Figure 10C**). These findings were robustly validated in TCGA-PRAD bulk RNA-seq cohort (**Figure 10D**). Furthermore, to explore IFRD1’s influence on the tumor microenvironment, we performed immune cell deconvolution analysis. High IFRD1 expression was significantly associated with increased infiltration of plasma cells, while rest mast cells and B cells were reduced, indicating that IFRD1 actively remodels the immune landscape toward a pro-tumorigenic state (**Figures 10E, F**). Integrated analysis revealed that IFRD1 expression correlates with a unique co-expression network, advanced pathology, altered immune infiltration, and poor prognosis as well as immunotherapy non-response across pan-cancer level (**Figure 10G**; **Supplementary Figure S4C–I**). This comprehensive profile suggests that IFRD1 functions not merely as a passive marker, but as an active transcriptional regulator and immune modulator, ultimately shaping clinical outcomes. To functionally validate the oncogenic role of IFRD1, we conducted *in vitro* experiments using the C4–2 PCa cell line. The efficiency of siRNA-mediated IFRD1 knockdown was first confirmed by qRT-PCR (**Figure 10H**). Functional assays demonstrated that IFRD1 knockdown significantly impaired cellular proliferation and suppressed migratory capacity (**Figures 10I, J**).

In summary, our multi-faceted investigation establishes IFRD1 as a critical mediator of UPR signaling in PCa, which drives tumor progression by coordinating oncogenic pathway activation and remodeling the tumor immune microenvironment. The compelling prognostic association and functional indispensability of IFRD1 position it as a promising therapeutic target for combating PCa.

## 4 Discussion

The UPR, functioning as a central integrator of endoplasmic reticulum stress, has been established as a critical mechanism intimately linked to tumor development and progression by driving malignant evolution across three fundamental axes: proteostasis remodeling, metabolic reprogramming, and immune evasion in multiple solid malignancies (7, 33). Nevertheless, the precise mechanisms through which UPR drives PCa progression, particularly its role in promoting tumor heterogeneity and therapeutic resistance, remain incompletely elucidated and warrant intensive investigation. Elucidating these mechanisms holds substantial significance for the discovery of clinically actionable stratification biomarkers and the development of precision therapeutic interventions (26).

Employing a three-tier hierarchical strategy encompassing single-cell resolution analysis, bulk-level modeling, and pivotal target validation, we integrated 1736 bulk transcriptomes with 36 scRNA-seq datasets to systematically dissect the role of UPR in prostate cancer through multi-omics approaches coupled with multiple machine-learning algorithms, thereby elucidating its intrinsic associations with intratumoral heterogeneity, tumor microenvironment remodeling, and clinical prognosis. We constructed a robust, machine learning-derived UPRRS based on UPRRGs and rigorously validated its predictive performance across multiple independent cohorts. Functional experiments further identified IFRD1 as a critical bottleneck node within the UPR network, providing an actionable entry point for clinical translation. Collectively, our study not only elucidates the pivotal role of UPR in prostate cancer progression but also provides a valuable predictive biomarker and proposes IFRD1 as a promising target for personalized therapeutic intervention.

Single-cell transcriptomic analysis revealed significant UPR activation within prostate epithelial cells, with the LE-KLK3 subpopulation exhibiting the most pronounced activity. This subpopulation displayed transcriptional profiles highly enriched for ER stress (34), hypoxia response, and androgen signaling, suggesting that UPR activation enhances cellular adaptability to stress through the regulation of protein homeostasis and metabolic reprogramming (35, 36). Notably, UPR-high cells showed no association with stemness or enhanced differentiation potential as assessed by CytoTRACE analysis (37), indicating that the UPR primarily promotes survival mechanisms rather than driving tumor initiation or dedifferentiation. Furthermore, UPR-high epithelial cells demonstrated enhanced communication with other components of the TME, acting as more active senders and receivers of signals in key pathways including TNF, VEGF, NOTCH, and IL6 (38). This suggests that the UPR not only mediates cell-autonomous stress adaptation but also may remodel the TME through paracrine signaling, thereby promoting angiogenesis, immunosuppression, and tumor cell invasion and metastasis. For the first time, our study pinpointed its spatial origin to the LE-KLK3 subpopulation, and revealed that it orchestrates the recruitment of CAFs and M2-TAMs to the tumor core via three distinct paracrine axes: the TNF→NF-κB axis, the hypoxia-VEGF axis, and the IL6→IL6ST axis, thereby establishing a proactive feedback circuit among UPR-high epithelial cells, CAFs, and immunosuppressive microenvironment, which provides a microecological framework for understanding persistent immune evasion in prostate cancer. Critically, UPR activity not only distinguished tumor from normal tissue but also stratified patients by aggressiveness (Gleason score, T stage) and outcomes (PFI, OS), reinforcing its translational relevance for risk stratification in clinical practice.

In this study, we developed a prognostic signature (UPRRS) derived from multiple machine learning algorithms based on UPRRGs, ultimately streamlining the model to seven pivotal genes. We subsequently validated the robust diagnostic and predictive performance of UPRRS across multiple independent cohorts, demonstrating its cross-platform and cross-ethnic robustness. Patients in the high-UPRRS group exhibited significantly shorter progression-free interval (PFI) and elevated expression of immune checkpoint molecules (including PDCD1 and CTLA4). Deconvolution analysis of immune infiltrates further revealed markedly enhanced intratumoral immunosuppressive signatures, characterized by increased infiltration of immunosuppressive cell populations such as regulatory T cells (Tregs) (10, 39). These findings suggest that UPR activation may foster an immunosuppressive TME and promote tumor progression by facilitating immune escape. This observation aligns with established literature indicating that the UPR can remodel the tumor immune microenvironment by modulating antigen presentation, cytokine secretion, and immune cell functions (40–42). In-depth interrogation of the immune microenvironment in UPRRS-stratified patients suggests that UPRRS can prospectively identify “immune-cold” tumors, providing a molecular basis for clinically selecting patients likely to benefit from immunotherapy.

At the level of clinical translation, the UPRRS showed significant correlations with established clinicopathological indicators of disease aggressiveness, including Gleason score, T stage, and lymph node metastasis. These associations further support the utility of the UPRRS as a robust prognostic tool. The clinical nomogram we developed, which integrates the UPRRS with T stage, demonstrated significantly improved predictive accuracy for survival, achieving an AUC of 0.81. The clinical practicality of this integrated model was confirmed by calibration curves and decision curve analysis, underscoring its promising potential for informing individualized treatment strategies. Drug sensitivity profiling further unveiled the clinical translational potential of UPRRS. Patients in the high-UPRRS cohort displayed a broader drug resistance profile across multiple chemotherapeutic agents, including docetaxel and carboplatin, compared to their low-UPRRS counterparts. This suggests that UPR activation may confer chemoresistance by enhancing cellular protein folding capacity, alleviating oxidative stress, and promoting DNA damage repair (43). Consequently, UPRRS can serve as a pretreatment molecular stratification decision-support tool, providing clinicians with a quantitative reference for selecting docetaxel- or platinum-based regimens and implying the potential synergistic value of combining UPR inhibitors with chemotherapy.

Collectively, UPRRS not only represents a cross-cohort robust diagnostic and prognostic biomarker but also functions as a dual-scenario stratification tool spanning chemotherapy and immunotherapy, providing a quantifiable molecular ruler for precision management of prostate cancer (44).

Among the seven constituent genes comprising UPRRS, IFRD1 emerged as the prime candidate for functional interrogation, attributed to its robust and consistent diagnostic-prognostic weight across pan-cancer, multi-cohort, and cross-platform analyses. Previous studies have established IFRD1 not only as a stress-responsive gene but also as a key promoter of tumor cell survival across various cancer types (45). Its mechanisms of action are intricately linked to the UPR, metabolic reprogramming, and immune evasion (45). In PCa, IFRD1 has been identified as a component of a 10-gene signature predictive of enzalutamide resistance. Its elevated expression correlates with shorter recurrence-free survival and is closely associated with cell cycle pathway activation (46). Our data confirm that IFRD1 is highly expressed across multiple PCa cohorts and is strongly associated with an adverse prognosis. Functional enrichment analysis indicated that high IFRD1 expression is significantly associated with pro-tumorigenic pathways, including UPR, ER stress, hypoxia, and EMT. This suggests that IFRD1 acts as a critical hub within the UPR signaling network, coordinating tumor cell survival, migration, and immune evasion (9, 47). Importantly, our study extends the understanding of IFRD1 function by demonstrating its profound impact on the immune microenvironment. Beyond prostate cancer, our pan-cancer analysis reveals that IFRD1’s prognostic significance is recapitulated in breast, head and neck, and cholangiocarcinoma cohorts. Moreover, its association with immunotherapy non-response highlights a potential role in mediating immune evasion across cancer types. This pan-cancer relevance substantially enhances the translational value of targeting IFRD1, particularly for patients with immunotherapy-refractory disease. *In vitro* functional assays further substantiated the oncogenic role of IFRD1. Knockdown of IFRD1 in PCa cells significantly suppressed proliferative and migratory capacities, supporting its potential as a viable therapeutic target. Future studies should aim to delineate the precise mechanisms by which IFRD1 regulates the UPR signaling axis and to characterize its dynamic expression and functional roles in response to various therapeutic pressures, including androgen deprivation therapy (ADT), chemotherapy, and immunotherapy.

### 4.1 Limitation of the study

Although this study systematically elucidated the prognostic value and therapeutic potential of UPR in prostate cancer through a three-tier integrated strategy spanning single-cell, bulk, and functional dimensions, this study has several limitations that should be acknowledged. First, our analyses are predominantly derived from retrospective analyses of public datasets and thus lack validation in prospective clinical cohorts. Second, although the UPRRS demonstrated robust predictive performance in the cohorts analyzed, its generalizability and clinical utility require further verification in larger, more diverse patient populations. Furthermore, while our *in vitro* findings support the oncogenic role of IFRD1, its mechanistic functions require further *in vivo* validation. We have therefore initiated drug discovery efforts to identify clinically applicable drugs that can inhibit IFRD1 or even modulate UPR activity. Finally, given that the UPR is a highly dynamic and context-dependent signaling network, its functional roles and the prognostic value of the UPRRS may vary under different therapeutic pressures, including ADT, radiotherapy, and immunotherapy. Consequently, future investigations should prioritize evaluating the predictive value of the UPRRS within specific treatment contexts.

## 5 Conclusion

In summary, we systematically implicated the molecular characteristics and clinical significance of UPR in PCa through multi-omics integrated analysis, constructed a UPRRS model with good predictive performance, and preliminarily verified its application value in prognosis assessment, tumor immune microenvironment analysis, and drug sensitivity prediction. IFRD1, as a key regulatory factor in the UPR signaling network, demonstrates potential as a therapeutic target. Our research provided new theoretical basis and tool support for the precision medicine of PCa, and is expected to be applied in clinical practice in the future.

## Data Availability

The original contributions presented in the study are included in the article/**Supplementary Material**. Further inquiries can be directed to the corresponding authors.

## Supplementary material

Supplementary Figure 1 UPR characteristics in single-cell transcriptome. **(A)** The Integration of thirteen primary PCa samples before. **(B)** Dot plot showing the marker genes expression for each cell type, where the dot size and color represent the percentage of marker gene expression and the averaged scaled expression value, respectively. **(C)** Heatmap showing the top 5 marker genes in each cell cluster. **(D)** Box plots showing the clinical relevance of UPR expression levels in the GSE116918, GSE54460, GSE70768 and TCGA-PRAD cohort. **(E)** Kaplan-Meier curves for OS and DSS of PRAD patients with high and low expression level of UPR in the TCGA-PRAD cohort.

Supplementary Figure 2 UPR activity drives transcriptomic divergence in PCa epithelial cells. **(A, B)** In the GSE141445 cohort, UMAP plots showing the distribution **(B)** of luminal cells related to the expression level of UPR **(A**, **C)** UMAP plots showing the eight cell types identified by marker genes as well as expression level of UPR in the GSE1414456 cohort. **(D)** GSEA functional enrichment analysis results of the hypoxia and androgen response pathway in the high UPR subgroup. **(E)** UMPA plots showing the CytoTRACE analyses results about the relative order and potency scores in luminal cells in the GSE141445 cohort. **(F)** GSEA functional enrichment analysis in the TCGA-PRAD database. **(G, H)** Bubble charts of Gene Ontology (GO) **(G)** and KEGG **(H)** functional enrichment analysis for DEGs between UPR-high and UPR-low subgroups in the TCGA-PRAD database.

Supplementary Figure 3 Identification of UPRRGs using WGCNA. **(A)** Heatmap showing the CellChat of incoming and outgoing signaling patterns in eight immune cells. **(B)** The determination of the optimal soft threshold in WGCNA analysis. **(C)** Heatmap showing the clustering relationship between gene modules and samples. **(D)** Heatmap of correlations between gene modules. **(E)** Box plot showing the expression levels of the seven pivotal genes obtained in stepwise Cox regression between normal and tumor tissues. **(F)** Random Forest Error Curve Chart. **(G)** Bar plot of log-rank test results for the seven genes from Random Forest analysis, with the x-axis representing MDA and MDG. * p < 0.05, ** p < 0.01, *** p < 0.001, **** p < 0.0001

Supplementary Figure 4 Immune cell landscape between high- and low-risk subgroups and research on genes co-expression with IFRD1. **(A)** Rainbow plot showing the proportions of 21 immune cell types calculated by CIBERSORT in high- and low-risk subgroups, with each bar representing a patient and each color representing an immune cell type. **(B)** Box plot showing the expression levels of immune cells between high- and low-risk subgroups. **(C)** Bar plots showing the distribution of clinical characteristics for different expression levels of IFRD1, including AGE, N stage, GS score and T stage. **(D)** Kaplan-Meier curves of patients with other tumors between high and low expression level of IFRD. **(E)** Box plot showing the difference of immunotherapy response between high and low expression level of IFRD in the GSE91061 cohort. **(F, G)** Heatmap showing the expression level of top 50 genes co-expressed with IFRD1, the positive expression **(F)** and the negative expression **(G)** of IFRD1 respectively. **(H, I)** Volcano plots of IFRD1 GO **(H)** and KEGG **(I)** pathways in TCGA-PRAD cohort.

## Glossary

PCa: Prostate cancer
PRAD: Prostate adenocarcinoma
CRPC: Castration-resistant prostate cancer
UPR: Unfolded protein response
WGCNA: Weighted gene co-expression network analysis
TME: Tumor microenvironment
ICB: Immune checkpoint blockade
ER: Endoplasmic reticulum
UPRRGs: Unfolded protein response-related genes
UPRRS: Unfolded protein response-related signature
RNA-seq: RNA sequencing
TCGA: The Cancer Genome Atlas
GEO: Gene Expression Omnibus
scRNA-seq: Single-cell RNA sequencing
DEGs: Differentially expressed genes
ssGSEA: Single-sample gene-set enrichment analysis
GSEA: Gene Set Enrichment Analysis
GO: Gene Ontology
KEGG: Kyoto Encyclopedia of Genes and Genomes
BP: Biological Process
MF: Molecular Function
CC: Cellular Component
TOM: Topological overlap matrix
K-M: Kaplan-Meier
ROC: Receiver Operating Characteristic
LASSO: Least Absolute Shrinkage and Selection Operator
AUC: Area under the curve
IPS: Immunophenoscore
TCIA: The Cancer Immunome Atlas
CIBERSORT: Cell-type Identification by Estimating Relative Subsets of RNA Transcripts
IC50: Half-maximal inhibitory concentration
RT-qPCR: Real-time quantitative reverse transcription polymerase chain reaction
IHC: Immunohistochemistry
FDR: False discovery rate
OS: Overall survival
PFI: Progression-free interval
PCA: Principal component analysis
GS: Gene significance
MM: Module membership
Mda: Mean decrease accuracy
Mdg: Mean decrease gini
IFRD1: Interferon-related developmental regulatory factor 1
EMT: Epithelial-mesenchymal transition
ADT: Androgen deprivation therapy
