# Supplementary figures and images for: Multi-omics analyses related to unfolded protein response in prostate cancer implicate pro-tumor role of IFRD1
Source: Front Immunol. 2026 Jan 29;17:1744197. doi: 10.3389/fimmu.2026.1744197 (PMC12894258; doi:10.3389/fimmu.2026.1744197)

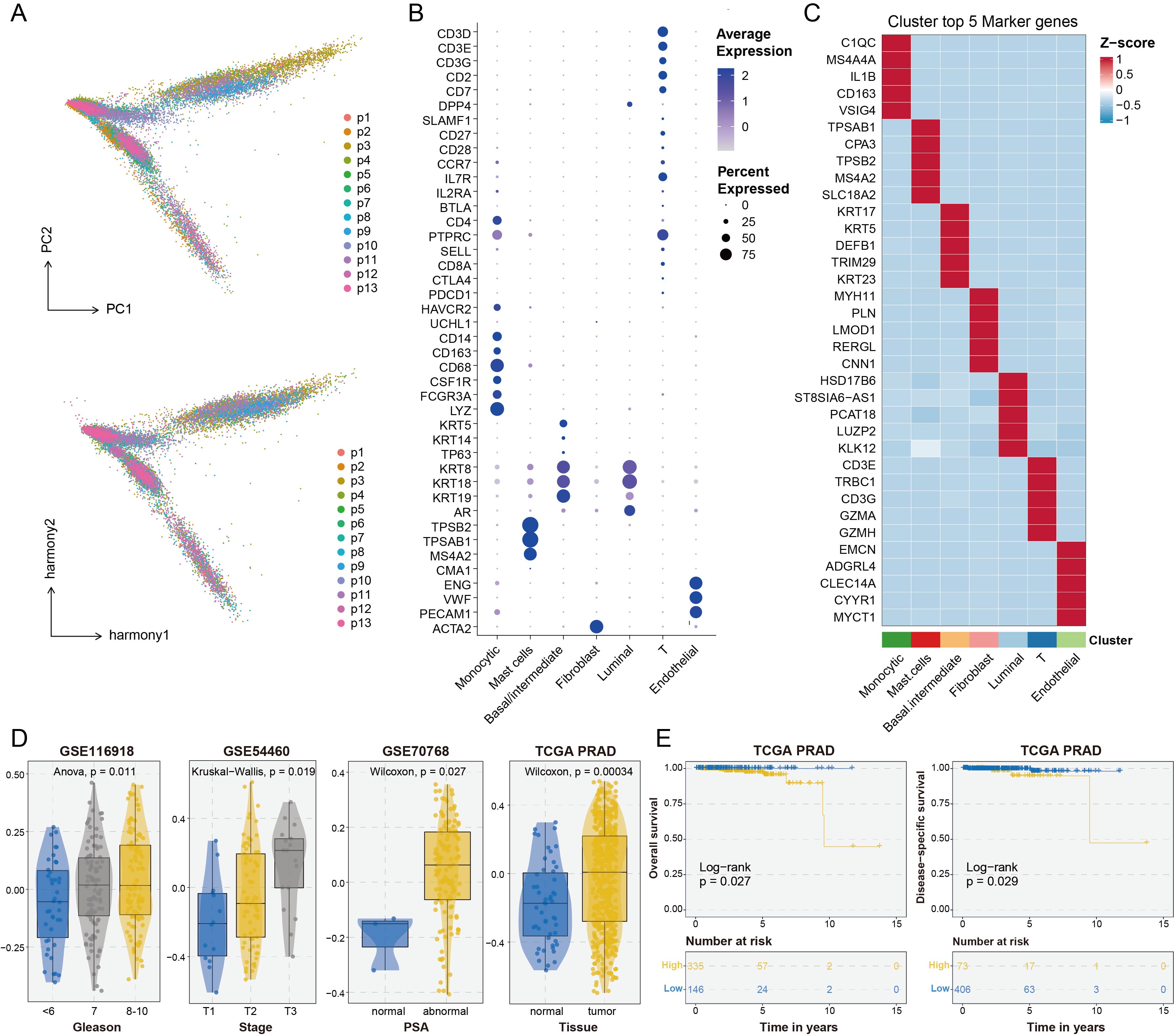

Supplement: Supplementary Figure 1 — UPR characteristics in single-cell transcriptome. (A) The Integration of thirteen primary PCa samples before. (B) Dot plot showing the marker genes expression for each cell type, where the dot size and color represent the percentage of marker gene expression and the averaged scaled expression value, respectively. (C) Heatmap showing the top 5 marker genes in each cell cluster. (D) Box plots showing the clinical relevance of UPR expression levels in the GSE116918, GSE54460, GSE70768 and TCGA-PRAD cohort. (E) Kaplan-Meier curves for OS and DSS of PRAD patients with high and low expression level of UPR in the TCGA-PRAD cohort. [file Image1.jpeg]

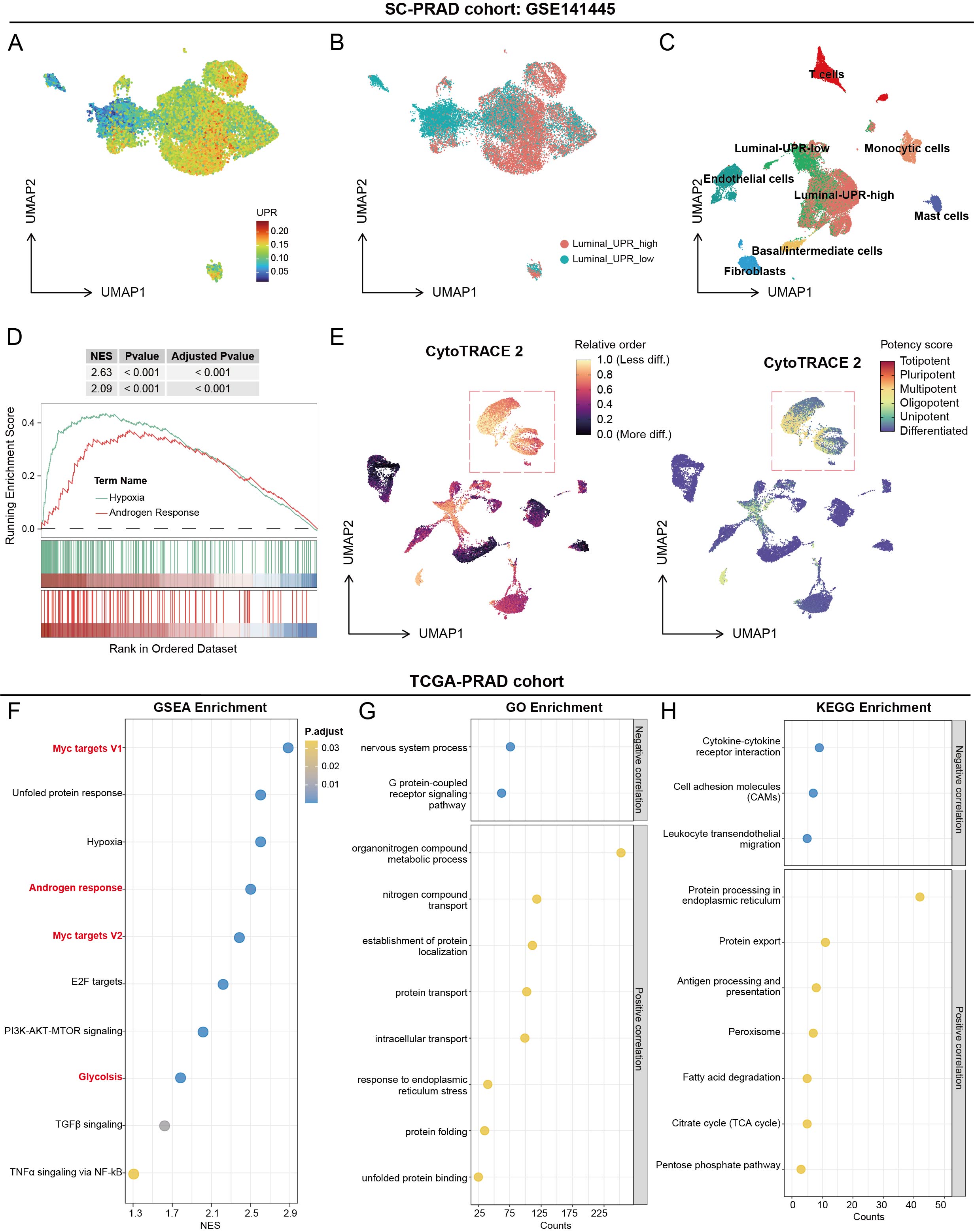

Supplement: Supplementary Figure 2 — UPR activity drives transcriptomic divergence in PCa epithelial cells. (A, B) In the GSE141445 cohort, UMAP plots showing the distribution (B) of luminal cells related to the expression level of UPR (A, C) UMAP plots showing the eight cell types identified by marker genes as well as expression level of UPR in the GSE1414456 cohort. (D) GSEA functional enrichment analysis results of the hypoxia and androgen response pathway in the high UPR subgroup. (E) UMPA plots showing the CytoTRACE analyses results about the relative order and potency scores in luminal cells in the GSE141445 cohort. (F) GSEA functional enrichment analysis in the TCGA-PRAD database. (G, H) Bubble charts of Gene Ontology (GO) (G) and KEGG (H) functional enrichment analysis for DEGs between UPR-high and UPR-low subgroups in the TCGA-PRAD database. [file Image2.jpeg]

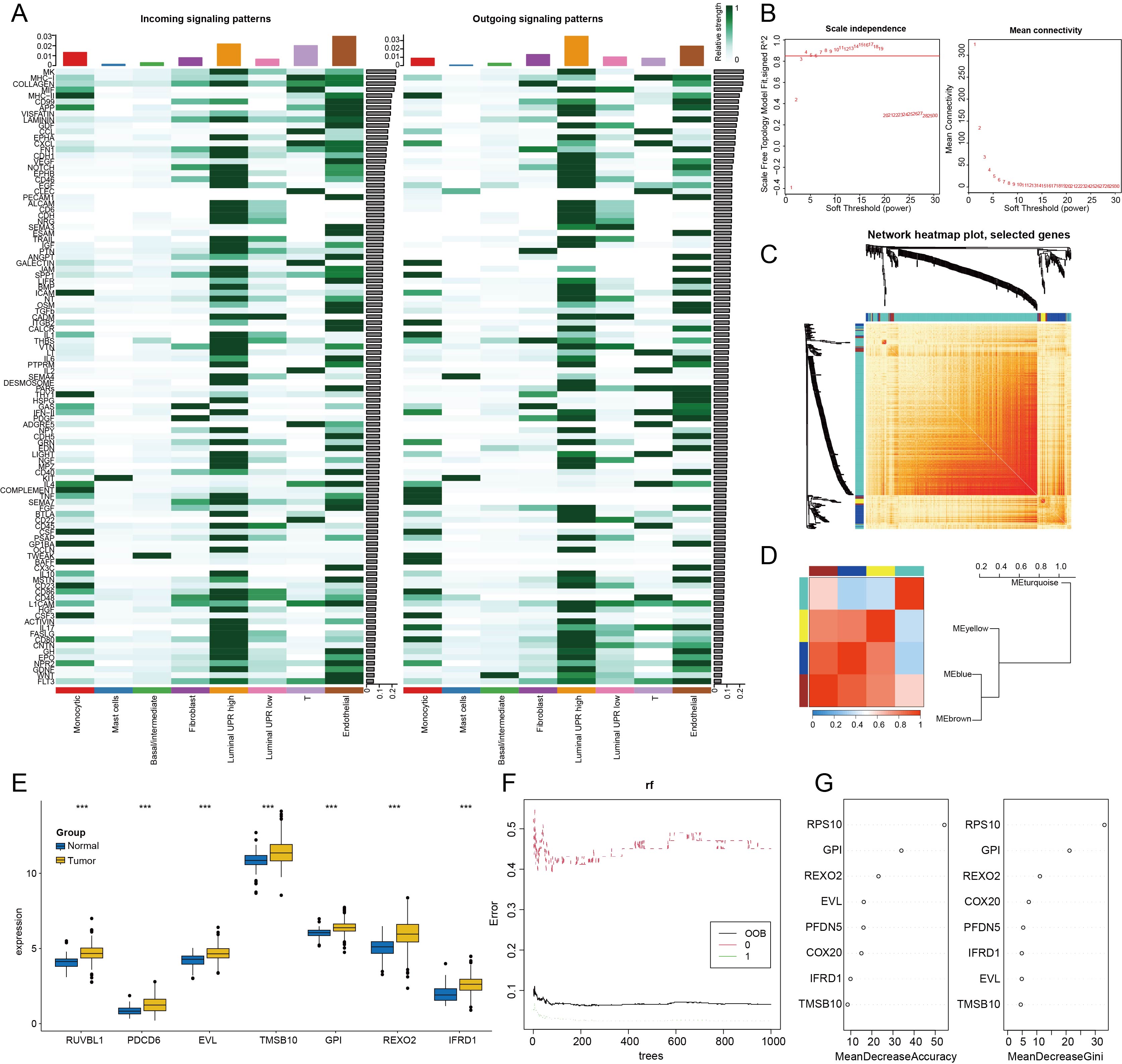

Supplement: Supplementary Figure 3 — Identification of UPRRGs using WGCNA. (A) Heatmap showing the CellChat of incoming and outgoing signaling patterns in eight immune cells. (B) The determination of the optimal soft threshold in WGCNA analysis. (C) Heatmap showing the clustering relationship between gene modules and samples. (D) Heatmap of correlations between gene modules. (E) Box plot showing the expression levels of the seven pivotal genes obtained in stepwise Cox regression between normal and tumor tissues. (F) Random Forest Error Curve Chart. (G) Bar plot of log-rank test results for the seven genes from Random Forest analysis, with the x-axis representing MDA and MDG. * p < 0.05, ** p < 0.01, *** p < 0.001, **** p < 0.0001 [file Image3.jpeg]

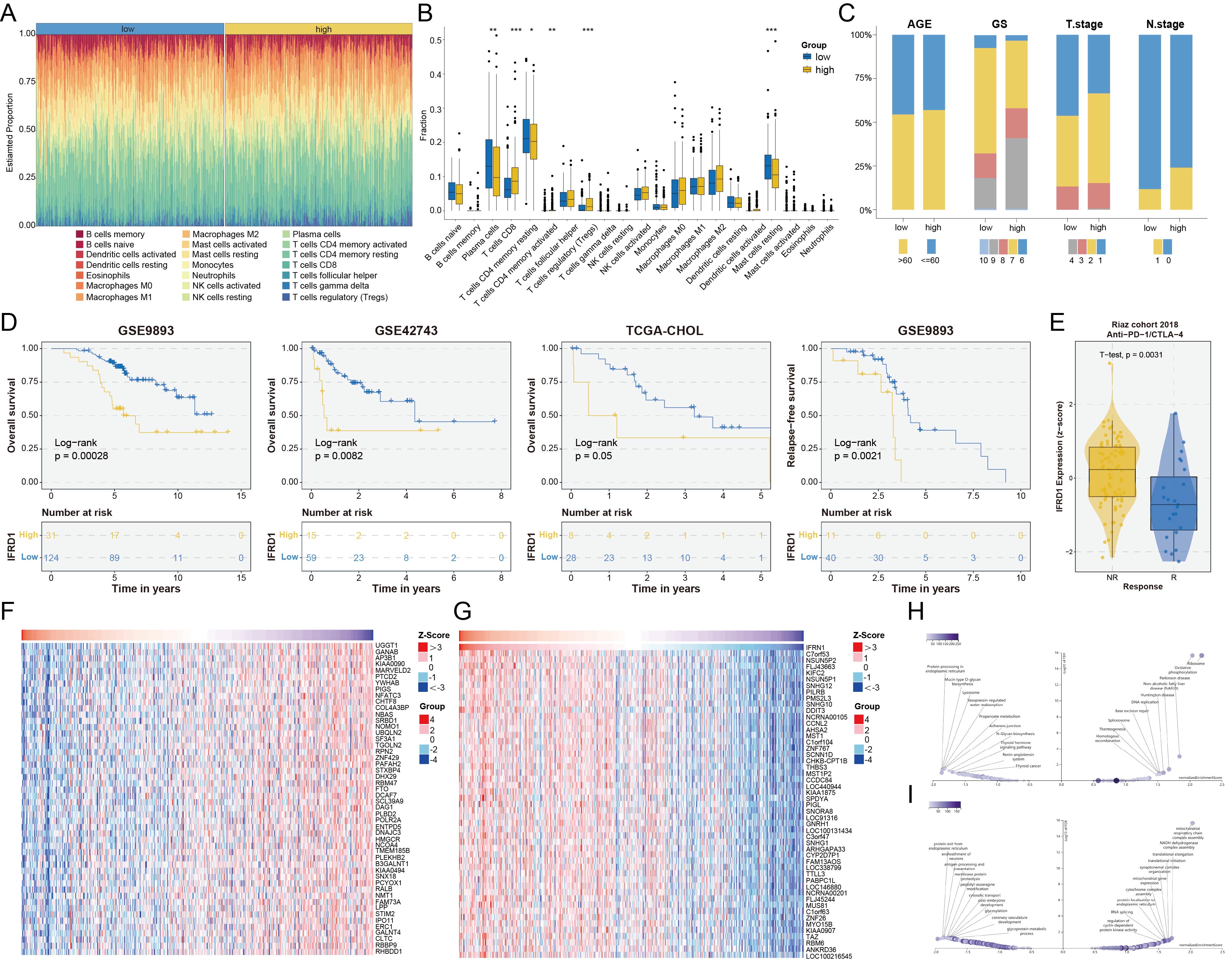

Supplement: Supplementary Figure 4 — Immune cell landscape between high- and low-risk subgroups and research on genes co-expression with IFRD1. (A) Rainbow plot showing the proportions of 21 immune cell types calculated by CIBERSORT in high- and low-risk subgroups, with each bar representing a patient and each color representing an immune cell type. (B) Box plot showing the expression levels of immune cells between high- and low-risk subgroups. (C) Bar plots showing the distribution of clinical characteristics for different expression levels of IFRD1, including AGE, N stage, GS score and T stage. (D) Kaplan-Meier curves of patients with other tumors between high and low expression level of IFRD. (E) Box plot showing the difference of immunotherapy response between high and low expression level of IFRD in the GSE91061 cohort. (F, G) Heatmap showing the expression level of top 50 genes co-expressed with IFRD1, the positive expression (F) and the negative expression (G) of IFRD1 respectively. (H, I) Volcano plots of IFRD1 GO (H) and KEGG (I) pathways in TCGA-PRAD cohort. [file Image4.jpeg]
